# Algorithms to reconstruct past indels: The deletion-only parsimony problem

**DOI:** 10.1371/journal.pcbi.1012585

**Published:** 2025-07-28

**Authors:** Jordan Moutet, Eric Rivals, Fabio Pardi

**Affiliations:** LIRMM, Université de Montpellier, CNRS, Montpellier, France; The Pennsylvania State University, UNITED STATES OF AMERICA

## Abstract

Ancestral sequence reconstruction is an important task in bioinformatics, with applications ranging from protein engineering to the study of genome evolution. When sequences can only undergo substitutions, optimal reconstructions can be efficiently computed using well-known algorithms. However, accounting for indels in ancestral reconstructions is much harder. First, for biologically-relevant problem formulations, no polynomial-time exact algorithms are available. Second, multiple reconstructions are often equally parsimonious or likely, making it crucial to correctly display uncertainty in the results. Here, we consider a parsimony approach where only deletions are allowed, while addressing the aforementioned limitations. First, we describe an exact algorithm to obtain all the optimal solutions. The algorithm runs in polynomial time if only one solution is sought. Second, we show that all possible optimal reconstructions for a fixed node can be represented using a graph computable in polynomial time. While previous studies have proposed graph-based representations of ancestral reconstructions, this result is the first to offer a solid mathematical justification for this approach. Finally we provide arguments for the relevance of the deletion-only case for the general case.

## Author summary

An exciting frontier in evolutionary biology is the ability to reconstruct DNA or protein sequences from species that lived in the distant past. By analyzing sequences from present-day species, we aim to infer the sequences of their common ancestors—a process known as ancestral sequence reconstruction. This task has far-reaching applications, such as resurrecting ancient proteins and studying the biology of extinct organisms. However, a significant challenge remains: the lack of well-established methods for inferring past deletions and insertions—mutations that remove or add segments of genetic code. In this paper, we present algorithms that lay the groundwork for addressing this gap. We show that finding the reconstructions involving only deletion events, while minimizing their number, can be done efficiently. Additionally, we show that all optimal solutions can be represented using specialized graphs. While previous studies have proposed graph-based representations of ancestral reconstructions, we are the first to provide a rigorous mathematical foundation for the use of these graphs.

## 1. Introduction

Biological sequences undergo mutations during evolution, and the most common ones are substitutions (when a nucleotide or amino acid is replaced by another), insertions (when a sequence is added inside another larger sequence), and deletions (when a part of a sequence is lost). Ancestral Sequence Reconstruction (ASR) is the task that consists in recovering the evolutionary history of a set of sequences related by a known phylogeny, that is the mutation events that explain the differences between these sequences. Finding where exactly these events occurred in the phylogeny amounts to determining the sequences that were present at the internal nodes of the phylogeny, that is, the *ancestral* sequences. ASR is a key task in bioinformatics, with wide-ranging applications, from protein engineering in biomedicine [1] and biotechnology [2], to protein resurrection for vaccine development [3] and paleontology [4], to the reconstruction of ancient genomes [5]. Additionally, ASR lies at the foundation of other tasks in bioinformatics, for example sequence alignment [6] and phylogenetic placement [7,8], meaning that any improvements to the methodology for ASR are likely to benefit these other subjects.

Polynomial-time algorithms to infer past substitutions were introduced decades ago, and now form a solid basis for ASR [9–14]. On the other hand, the inference of indels (insertions and deletions), despite multiple methodological attempts, does not have an established theoretical basis [6]. The lack of broadly adopted solutions for indels compared to substitutions can be explained by multiple factors. First, no exact algorithms that are polynomial both in the number of sequences and their length are known to solve the most biologically-relevant formulations of this problem. Thus, some algorithms are exact but have worst-case running times exponential either in the number of sequences [15–17] or their length [18,19]. Usually, the available polynomial-time algorithms are heuristic [5,16,20], or they are based on simplified modeling approaches such as indel coding and its variants [21–23] or the assumption of site independence [24,25]. Another challenge for the inference of past indels is that multiple reconstructions are often equally parsimonious/likely, meaning that it can be misleading to just return a single optimal solution. Instead, it is important to provide some insight about the other reconstructions. Exploring the diversity of solutions not only carries an additional computational cost, but it also poses the problem of how to correctly display uncertainty in the results. Even though this issue is also present for the inference of past substitutions, it is much more problematic for indels, as they can overlap each other. A graph-based solution to represent multiple reconstructions for a single node in the phylogeny, showing multiple alternative starting and ending positions for each putative gap, was recently put forward by [23]. Despite the problems outlined above, indel inference shows a rebirth of interest, with two parsimony-based works published in the past year [25,26].

Similarly to substitutions, indels can be inferred using two broad approaches. The conceptually simpler one is maximum parsimony. Here, a number of formulations of the problem were already proposed two decades ago [15,18,27]. Although similar in spirit, these approaches differred in terms of some important modeling choices (e.g. on how to count indels between two sequences, and on whether to allow multiple introductions of the same character). The formulation by [27] is arguably the most faithful to the biological context, and was later adapted into number of maximum-likelihood approaches for indel inference [16,28,29]. It leads to a problem called the Indel Parsimony Problem (IPP), which we formally define below (Definition 5). Although the IPP is NP-hard for trees of unbounded degree, its complexity remains unknown for trees with bounded degree, in particular binary trees, and under the unit-cost model, consisting of minimizing the number of indels irrespective of their size or place in the tree.

The second broad approach for indel inference is the statistical one. In practice one assumes a probabilistic model explicitly describing the evolution of indels (e.g. [30–34]) and then seeks the evolutionary history or histories with highest probability [16,19,29,35]. This idea can be formulated either as an optimization problem or in a Bayesian context [24,36]. For many tasks in phylogenetics, maximum parsimony has a computational advantage over statistical methods, which may rapidly become considerable as the number of sequences grows [37,38]. It is reasonable to expect a similar advantage for indel inference. Moreover statistical approaches may benefit from the insights gained from studying the algorithmics of maximum parsimony (see, e.g., the similarities between Sankoff’s and Felsenstein’s algorithms [10,39]).

In this paper, we focus on the specific case of parsimony-based ASR where solutions are constrained to only involve deletions. This problem, which we call the *deletion-only parsimony problem* (DPP), was first introduced by [27]. As we discuss more extensively in Sect 7, the DPP is relevant to the solution of the general problem (the IPP mentioned above), as parts of the IPP can be optimally solved alongside this problem. The first contribution of this paper is the proposal of an exact algorithm to find all optimal solutions of the DPP. Since the number of solutions may grow exponentially in the size of the input alignment, our algorithm will also run in exponential time. However, it is easy to modify the algorithm so that it can find a single optimal solution in polynomial time. Naturally, we formally prove the correctness of this algorithm (i.e. that all, and only, the optimal solutions of the DPP are found). Secondly, we propose a polynomial alternative for the exponential-time part of the algorithm, constructing a graph for each internal node of the phylogenetic tree. Each of these graphs represents exactly the set of all reconstructions that can be found at the specified node in an optimal solution for the whole tree. These graphs are similar to the ones constructed by [23] and [26], also known as *partial order graphs* (POGs; [40]). We discuss the relation with these and other recent works in Sect 8.

After this introduction, Sect 2 settles the notations and definitions that are necessary to introduce the IPP and the DPP. Then, Sect 3 describes our algorithm, and illustrates it step by step with a recurring example. Sect 4 presents the theoretical results about the algorithm’s correctness. Then, in Sect 5, we describe and illustrate our graph-based representation of optimal solutions, and the algorithm to construct it. Afterwards, in Sect 6, we analyze the asymptotic complexity of all the provided algorithms. Sect 7 deals with the issue of the relevance of the DPP for the solution of the IPP, and, finally Sect 8 concludes with some remarks about our original contributions, and the questions left open. The proofs of all our mathematical statements and a number of other accessory results are given in S1 Text.

## 2. Preliminaries

### 2.1. Basic notation

**Notation for phylogenetic trees.** We define a *(phylogenetic) tree T* as a binary rooted tree, whose root, set of edges, set of nodes and set of leaves are denoted by r(T),E(T),V(T) and *L*(*T*), respectively. Given two nodes u,v∈V(T), we say that *v* is a *descendant* of *u* and that *u* is an *ancestor* of *v* if the path from *r*(*T*) to *v* traverses *u*; in particular, if v≠u we say that *v* is a *strict descendant* of *u*, and that *u* is a *strict ancestor* of *v*. *T*_*u*_ denotes the subtree of *T* induced by the set of descendants of *u*. Finally, if (p,w)∈E(T) and *w* is a descendant of *p*, we say that *p* is the *parent* of *w*, and that *w* is a *child* of *p*.

**Notation and assumptions for alignments.** Given a set *X*, a (*multiple sequence*) *alignment A for X* is a table whose columns are labeled by a set of consecutive integers, called *sites*, and whose rows are in a one-to-one correspondence with the elements of *X*, called *taxa*. In particular, given x∈X and site *i*:

- *A*_*x*_ denotes the row of *A* corresponding to *x*;
- *A*[*i*] denotes the column *i* of *A*;
- $A_{x}[i]$ denotes the element of *A* at column *i* and at the row corresponding to *x*.

Although in general $A_{x}[i]$ may be an element of any fixed alphabet, throughout this paper we assume *A* has been preprocessed so as to be binary, i.e. $A_{x}[i]\in \{0,1\}$, where 1 and 0 are used to represent presence and absence of a character at site *i*, respectively. For any row *A*_*x*_ of an alignment, $𝟙(A_{x})$ denotes the set of sites that are set to 1 (i.e. filled), that is $𝟙(A_{x})=\{k:A_{x}[k]=1\}$.

For any alignment in input, we also assume that it has been preprocessed by adding a first column *A*[0] and a last column *A*[*m* + 1], with both *A*[0] and *A*[*m* + 1] only containing 1s, and where *m* is the number of columns in the original alignment. Finally we assume that no column *A*[*i*] contains only 0s.

**Notation about intervals and gaps.** Given two integers *i* and *j*, the set of all integers *k* with i≤k≤j is denoted by [i,j] and is called an *interval*. Two intervals [i,j] and $\left[i^{\prime },j^{\prime }\right]$ are said to *overlap* if $[i,j]\cap [i^{\prime },j^{\prime }]\neq \emptyset$; in particular, if $\left[i,j]⊈[i^{\prime },j^{\prime }\right]$ and $\left[i,j]⊉[i^{\prime },j^{\prime }\right]$, then [i,j] and $\left[i^{\prime },j^{\prime }\right]$ are said to *partially overlap*. Moreover, $A_{x}[i,j]$ denotes the sequence of characters at row *x* in alignment *A* between (and including) sites *i* and *j*.

**Definition 1** (*gap*). Let [i,j]⊆[1,m]. $A_{x}[i,j]$ is a gap if all characters in $A_{x}[i,j]$ are 0s and $A_{x}[i-1]=A_{x}[j+1]=1$.

### 2.2. Alignment extensions and indels

Alignments encountered in practice usually only contain information for the leaves of a phylogenetic tree and are thus alignments for *L*(*T*) where *T* is an unknown tree. Given a putative tree *T* and an alignment for *L*(*T*), the goal of ancestral indel reconstruction is to recover the pattern of gaps at the internal nodes of *T*. This idea underlies the following definitions.

**Definition 2** (*alignment extension*). Let *T* be a tree and *A* an alignment for *L*(*T*). An alignment extension of *A* for *T* is an alignment *A*^ + ^ for *V*(*T*), such that $A_{v}^{+}=A_{v}$ for any v∈L(T).

**Definition 3** (*phylogenetic correctness*). An alignment extension *A*^ + ^ for *T* is said to be phylogenetically correct if for any two nodes u,v∈V(T) and any site *i*, such that $A_{u}^{+}[i]=A_{v}^{+}[i]=1$, we have $A_{x}^{+}[i]=1$ for each node *x* in the path between *u* and *v*.

The biological intuition behind phylogenetic correctness is the following. If two nodes u,v satisfying $A_{u}^{+}[i]=A_{v}^{+}[i]=1$ were separated by a node *x* with $A_{x}^{+}[i]=0$, that would imply that either the character at site *i* has been introduced by two separate insertion events, or that it has first been lost with a deletion and then re-introduced with an insertion. Both of these scenarios contradict the phylogenetic interpretation of alignments, in which two characters should only be placed at the same site in an alignment if they derived from the same ancestral character. For these reasons, the problems that we consider in this paper (Definitions 5 and 6 below) constrain alignment extensions to be phylogenetically correct. This constraint is equivalent to the common interpretation of “Dollo’s law” in phylogenetics, which asserts that no (complex) character can be introduced more than once during the course of evolution [41]. Recent works impose phylogenetic correctness by presupposing Dollo’s law [25].

The last missing ingredient before we can introduce the problems to solve is a definition of insertions and deletions, and how to count them. In a parsimony context, this can be done as in Definition 4, which is equivalent to the one by [27].

**Definition 4** (*insertion, deletion, unit-cost distance*). Let *T* be a tree and (u,v)∈E(T). Let *A* be an alignment for X⊇{u,v}. We say that *A* has a *deletion* from *u* to *v* at interval [i,j] if the following statements are all verified

1.
$\left(A_{u}[i],A_{v}[i])=(1,0\right)$

2.
$\left(A_{u}[j],A_{v}[j])=(1,0\right)$

3.
$\left(A_{u}[k],A_{v}[k])\neq (1,1),\forall k\in [i,j\right]$

4. [i,j] is maximal among all intervals satisfying the three points above.

Similarly, we say that *A* has an *insertion* from *u* to *v* at interval [i,j] if there is a deletion from *v* to *u* at [i,j]. Together, insertions and deletions are called *indels*. Sites *i* and *j* are called the *endpoints* of the indel. The number of indels from *u* to *v* is also called the *unit-cost distance* between *u* and *v* and here will be noted as $d(A_{u},A_{v})$.

**Example 1.** Let *A*_*u*_ and $A_{v}$ be defined as follows:


$$\begin{array}{lllllllll} & 0 & 1 & 2 & 3 & 4 & 5 & 6 & 7\\ u: & 1 & 1 & 0 & 0 & 1 & 1 & 1 & 1\\ v: & 1 & 0 & 0 & 1 & 0 & 1 & 0 & 1 \end{array}$$

Here we have $d(A_{u},A_{v})=3$, and more specifically a deletion at [1,4], an insertion at {3}=[3,3], and a deletion at {6}=[6,6]. We use this example to illustrate the biological appropriateness of Definition 4, supported by a few informal arguments.

First, an interesting feature is that insertions and deletions can occur at overlapping intervals (e.g. [1,4] and [3,3] here). Indeed, other ways to define and count indels may result in considering the character losses at sites 1 and 4 as two separate deletions, implying a number of indels larger than 3 in this example [15,17,29]. However, this seems unjustified in a maximum parsimony context: we can imagine that at an intermediate node *x* (subdividing edge (u,v) into (u,x) and (x,v)) we may have $A_{x}=1\;0\;0\;0\;0\;1\;0\;1$, which can be explained with a single deletion acting on both sites 1 and 4 simultaneously from *u* to *x* (this is possible because sites 1 and 4 contain consecutive characters in the sequence at *u*). Additionally *A*_*x*_ implies a deletion at site 6 from *u* to *x* and an insertion from *x* to *v* at site 3. The existence of a scenario employing only 3 indels implies that, under maximum parsimony, $d(A_{u},A_{v})\leq 3$.

Moreover, any definition resulting in a number of indels smaller than 3 is also biologically implausible: since from *u* to *v* some characters are lost (at sites 1, 4, 6) and some other characters are gained (site 3) there must be at least one deletion and one insertion. Let us first assume that this is possible and that the insertion occurs *before* the deletion. Then the single deletion would have to simultaneously erase sites 1, 4, 6 without erasing intermediate sites 3 and 5. This is biologically unrealistic, as a single deletion should only be allowed to erase a set of consecutive characters. Let us now assume the opposite scenario, where the insertion occurs *after* the deletion. Since the deletion must remove the characters at sites 1 and 6 and all characters that are present in *u* between them, this would result in the loss of the character at site 5, followed by its re-introduction. As argued for phylogenetic correctness, this should not be allowed to happen under a biologically-relevant modelisation of indels (see paragraph following Definition 3).

### 2.3. Problem definition

Chindelevitch at al. [27] introduced the following problem and showed that it is NP-complete on trees of unbounded degree.

**Definition 5** (indel parsimony problem).

*Input:* A tree *T* and an alignment *A* for *L*(*T*).

*Output: A*^ + ^, a phylogenetically correct extension of *A*, minimizing the cost


$$c(A^{+})=\sum_{\left(u,v)\in E(T\right)}d(A_{u}^{+},A_{v}^{+}).$$

Here, we focus on a variant of this problem, where insertions are not allowed. We do this by specifying an additional constraint on *A*^ + ^:

**Definition 6** (deletion-only parsimony problem – DPP).

*Input:* A tree *T* and an alignment *A* for *L*(*T*).

*Output: A*^ + ^, an alignment extension of *A* minimizing *c*(*A*^ + ^) under the constraint that, for all (p,w)∈E(T) where *p* is the parent of *w*, all indels from *p* to *w* must be deletions.

**Fig 1:**
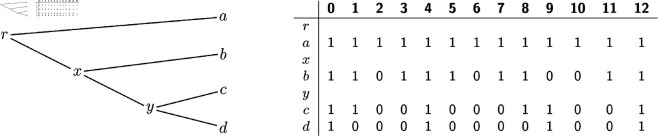
Example input of the DPP: a phylogenetic tree T and an alignment A for L(T).

**Fig 2:**
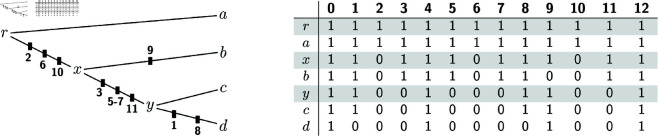
(Right:) A candidate solution *A*^ + ^ for the example in Fig 1. The rows shaded in grey are those that have been added when going from the input *A* to the extension *A*^ + ^. They correspond to the internal nodes of *T*. (Left:) The *c*(*A*^ + ^) = 9 deletions in *A*^ + ^ are here depicted as black boxes appearing on the edges where the deletions occur, along with their endpoints. The relative order of these boxes on their edge is uninformative.

We will refer to the extensions *A*^ + ^ that satisfy the constraint of the DPP as the *candidate solutions* of the DPP. Figs 1 and 2 show an example of the input and of a candidate solution of the DPP, respectively. We note that the candidate solution in Fig 2 is not optimal for the DPP, as we will show that there exist several solutions with a strictly lower *c*(*A*^ + ^).

**Observation 1.** Let *A*^ + ^ be a candidate solution of the DPP. Then, for every node *w* and site *k*:

1. If $A_{w}^{+}[k]=0$ then $A_{u}^{+}[k]=0,\forall u\in V(T_{w})$.
2. If $A_{w}^{+}[k]=1$ then $A_{u}^{+}[k]=1,\forall u$ in the path between *r*(*T*) and *w*.
3. *A*^ + ^ is phylogenetically correct.
4. $A_{r(T)}^{+}[k]=1$.

Points 1, 2 in Observation 1 are due to the fact that, because insertions are not allowed, no site *k* can be such that $A_{p}^{+}[k]=0$ and $A_{w}^{+}[k]=1$ for any p,w where *p* is the parent of *w*. Point 3 follows from the fact that any violation of phylogenetic correctness would also imply the existence of one such site. Finally, point 4 is due to the fact that if $A_{r(T)}^{+}[k]=0$, then a column of the input alignment *A* would only consist of gaps (0s).

## 3. The *deletion-only* algorithm

We now describe an algorithm that solves the DPP (Definition 6). The algorithm is designed to find not just one optimal solution, but all of them.

**High-level description.** Our algorithm for the DPP consist of two phases. The first phase is a bottom-up procedure, starting from the leaves and proceeding up to the root of the tree, conveying information about the presence of gaps in the descendants of each node. By doing so, we can determine some gaps that are necessarily present in every optimal solution (called 0-gaps, see next paragraph), and take note of the parts that can only be determined later on. The second phase is a top-down procedure, from the root to the leaves. It allows us to resolve the parts undetermined during the first phase, sometimes with multiple choices, providing a way to generate every possible optimal solution. Algorithm 1 provides the pseudo code for our algorithm, where procedures BottomUp and TopDown are defined further below (Algorithms 2 and 3 respectively).

**Algorithm 1. DeletionOnly(*T*, *A*).**



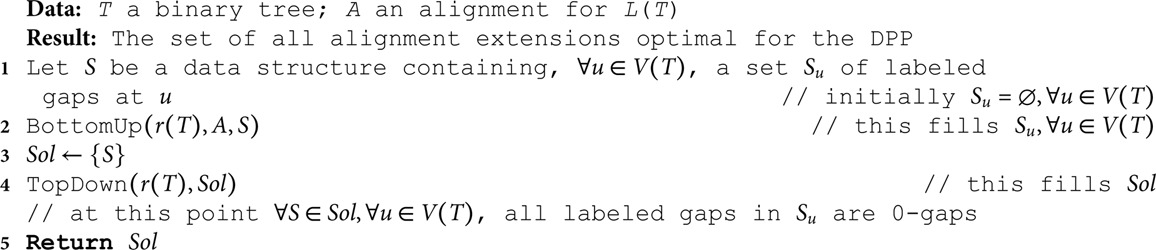



**Algorithm-specific notation.** For each node we store a set of *labeled gaps* defined as intervals [i,j]⊆[1,m] labeled with one of three symbols: 0, C or P. If an interval labeled by 0, C, P we call it *0-gap*, *C-gap* or *P-gap*, respectively. We explain the choice of this naming later below. If a 0-gap [i,j] is stored for node *u*, we also say that *u* has a 0-gap [i,j] or that [i,j] is a 0-gap at *u*. The same applies to C-gaps and P-gaps.

Remark that labeled gaps are those stored by the algorithm during its execution, and should not be confused with the gaps *tout-court* which are those that can be observed in an alignment, such as the input *A* or the output *A*^ + ^ (Definition 1).

**Algorithm 2. BottomUp(*w, A, S*).**



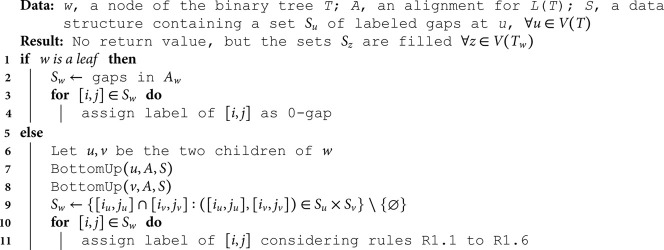



**Bottom-up phase.** We now describe how labeled gaps are created by the algorithm for every node *w*, in a bottom-up fashion. Algorithm 2 provides a recursive implementation of the bottom-up phase. If *w* is a leaf, its labeled gaps are set to the maximal intervals of sites filled with 0s in *A*_*w*_ and labeled as 0-gaps. In the example of Fig 1, leaf *d* has three 0-gaps: [1,3], [5,8] and [10,11].

Now let *w* be an internal node and *u* and *v* its children. For every pair of overlapping labeled gaps $\left[i_{u},j_{u}],[i_{v},j_{v}\right]$ at *u* and *v*, respectively, we store the labeled gap $\left[i,j]=[i_{u},j_{u}]\cap [i_{v},j_{v}\right]$ for node *w* and set its label with one of the following rules.

R1.1 If $\left[i_{u},j_{u}]=[i_{v},j_{v}\right]$ and neither of them is a P-gap at *u* and *v*, then [i,j] is labeled as a 0-gap at *w*.
R1.2 If $\left[i_{u},j_{u}]=[i_{v},j_{v}\right]$ and only one of them is a P-gap at *u* or *v*, then [i,j] is labeled as a C-gap at *w*.
R1.3 If $\left[i_{u},j_{u}]=[i_{v},j_{v}\right]$ and both are P-gaps at *u* and *v*, then [i,j] is labeled as a P-gap at *w*.
R1.4 If $\left[i_{u},j_{u}]⊊[i_{v},j_{v}\right]$ and $\left[i_{u},j_{u}\right]$ is not a P-gap at *u*, then [i,j] is labeled as a C-gap at *w*. The same applies if the roles of *u* and *v* are reversed.
R1.5 If $\left[i_{u},j_{u}]⊊[i_{v},j_{v}\right]$ and $\left[i_{u},j_{u}\right]$ is a P-gap at *u*, then [i,j] is labeled as a P-gap at *w*. The same applies if the roles of *u* and *v* are reversed.
R1.6 If $\left[i_{u},j_{u}\right]$ and $\left[i_{v},j_{v}\right]$ partially overlap, then [i,j] is labeled as a P-gap at *w*.

These rules cover every case and are exclusive. In fact, R1.1, R1.2, R1.3 obviously cover all equality cases, R1.4 and R1.5 cover the strict inclusion cases, and finally R1.6 covers the partial overlap case. Table 1 displays these labeling rules as a function of the labels of $\left[i_{u},j_{u}\right]$, $\left[i_{v},j_{v}\right]$, and of whether the two gaps are identical, strictly included in one another, or partially overlapping.

**Table 1:** Depiction of how the label of $\left[i,j]=[i_{u},j_{u}]\cap [i_{v},j_{v}\right]$ is assigned as a function of whether $\left[i_{u},j_{u}],[i_{v},j_{v}\right]$ are identical (row =), nested (rows ⊊,⊋), or partially overlapping (row “else”), and as a function of the labels of $\left[i_{u},j_{u}\right]$ (*u* columns) and of $\left[i_{v},j_{v}\right]$ (*v* columns).

	*u*	*v*	*u*	*v*	*u*	*v*
	0,C	0,C	0,C	P	P	P
=	0		C		P	
⊊	C		C		P	
⊋	C		P		P	
else	P		P		P	

The rationale for these rules is to guarantee the following properties. If [i,j] is a 0-gap at *w*, it means that *w* is either a leaf with this gap, or if we let u,v be the children of *w*, that there exists at least one leaf in *T*_*u*_ and at least one leaf in $T_{v}$ such that [i,j] is a gap at those leaves. If [i,j] is a C-gap, it means that only one (but not the other) of *T*_*u*_ and $T_{v}$ has some leaf with the same gap. Finally, if [i,j] is a P-gap at *w*, it means that none of the leaves that descend from *w* have this same gap. See Lemma 3 in S1 Text for a proof.

Labeled gaps also have implications about an optimal solution *A*^ + ^ of the DPP, as shown by a few propositions stated in the next section: Proposition 1 shows that if a site *k* does not belong to any labeled gap at *w*, then we must have $A_{w}^{+}[k]=1$. Proposition 2 shows that if [i,j] is a 0-gap at *w*, then $A_{w}^{+}[i,j]$ must be a gap; Proposition 3 shows that if [i,j] is a P-gap at *w*, then $A_{w}^{+}[i,j]=A_{p}^{+}[i,j]$ where *p* is *w*’s parent (hence the use of letter P for “parent”); finally Proposition 4 shows that if [i,j] is a C-gap at *w*, then either $A_{w}^{+}[i,j]$ must be a gap, or $A_{w}^{+}[i,j]=A_{p}^{+}[i,j]$ (hence letter C for “choice”).

**Example for the bottom-up phase.** At the end of the bottom-up phase, the example shown in Fig 1 produces the following labeled gaps at the internal nodes of the input tree.

Node *y* has two C-gaps, [2,3] and [5,7], both obtained by application of rule R1.4 on two 0-gaps at *c* and *d* (the children of *y*) with one 0-gap strictly contained in the other. Node *y* also has a 0-gap [10,11] obtained by application of rule R1.1 on two identical 0-gaps at *c* and *d*.

Node *x* has two C-gaps, [2,2] and [6,6], both obtained by application of rule R1.4 on a 0-gap at *b* strictly contained in a C-gap at *y*. Node *x* also has a P-gap [10,10] obtained by application of rule R1.6 on two partially overlapping 0-gaps at *b* and *y*.

**Fig 3:**
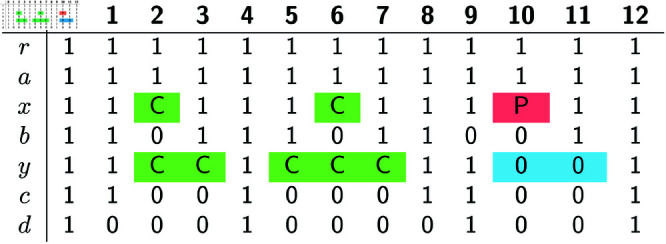
A depiction of the labeled gaps constructed by the bottom-up phase on the input shown in Fig 1. Note that Algorithm 2 does not store labeled gaps in this form.

Finally, *r* has no labeled gap, as no two labeled gaps at *a* and *x* overlap. The labeled gaps listed above are depicted in Fig 3, although we remark that the algorithm does not need to store labeled gaps in table form.

**Top-down phase.** The goal of the second phase is to remove C-gaps and P-gaps and replace them with one or more 0-gaps. These 0-gaps, together with those already determined during the bottom-up phase, will constitute the actual gaps in the output. As we explain below, sometimes (namely when applying rule R2.2), multiple optimal choices are possible, in which case we duplicate the current solution and carry on.

Algorithm 3 provides a recursive implementation of this phase in full detail, where intermediate solutions (those still containing C-gaps and P-gaps) are represented using a data structure *S* (e.g. a map) that allows to retrieve a set *S*_*w*_ of labeled gaps for each node *w*. We visit every node of the tree in pre-order. It is easy to see that the root *r*(*T*) cannot have any labeled gaps, because otherwise the input alignment would have one or more columns only containing 0s. (See Corollary 2 in S1 Text.) Thus, no action is needed for *r*(*T*). For every other node *w*, we process each of its C-gaps and P-gaps using one of the rules R2.1 to R2.3 below, where *p* denotes the parent of *w*.

R2.1 If [i,j] is a C-gap at *w*, and [i,j] is a 0-gap at *p*, then [i,j] is reset as a 0-gap at *w*.
R2.2 If [i,j] is a C-gap at *w*, and [i,j] is not a 0-gap at *p*, we duplicate the current solution. In the original solution we reset [i,j] as a 0-gap at *w*. In the duplicate, we copy at *w* all 0-gaps that are in [i,j] at *p*. That is, for each 0-gap $\left[i^{\prime },j^{\prime }]\subseteq [i,j\right]$ at *p*, we set $\left[i^{\prime },j^{\prime }\right]$ as a 0-gap at *w*, and [i,j] is removed from the set of labeled gaps at *w*.
R2.3 If [i,j] is a P-gap at *w*, then we copy at *w* all 0-gaps that are in [i,j] at *p*. That is, for each 0-gap $\left[i^{\prime },j^{\prime }]\subseteq [i,j\right]$ at *p*, we set $\left[i^{\prime },j^{\prime }\right]$ as a 0-gap at *w*. Moreover, [i,j] is removed from the set of labeled gaps at *w*.

As a result of the repeated application of these rules, in the end every stored solution is encoded as a collection of 0-gaps. To produce an alignment extension *A*^ + ^ it suffices to set every site within a 0-gap to 0 and every other site to 1.

We remark that every time we copy at *w* all 0-gaps that are in [i,j] at *w*’s parent *p*, this has the effect of setting $A_{w}^{+}[i,j]=A_{p}^{+}[i,j]$. This is a consequence of the fact that no two labeled gaps $\left[i_{w},j_{w}\right]$, $\left[i_{p},j_{p}\right]$ at *w* and *p* respectively, can be partially overlapping (see Lemma 1 in S1 Text).

**Algorithm 3. TopDown(*w, Sol*).**



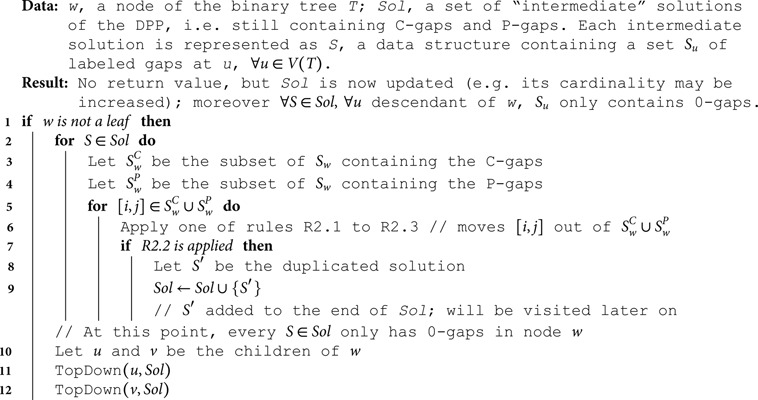



**Fig 4:**
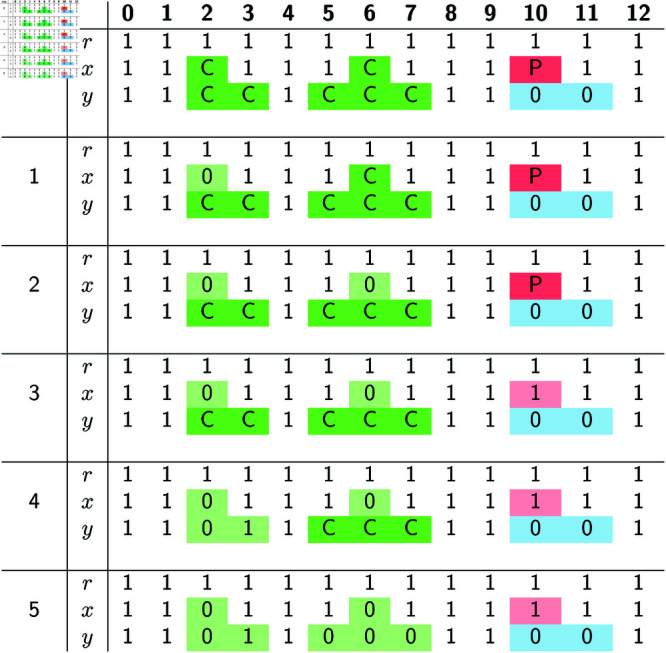
A sequence of intermediate solutions produced by the top-down phase on the input of Fig 1, leading to an optimal solution (step 5). Each solution is represented as an alignment restricted to the internal nodes *r*, *x* and *y*, even though the algorithm does not store labeled gaps in this form. Colored rectangles indicate sites covered or previously covered (lighter color) by labeled gaps. At step 0, no labeled gap has been processed and the intermediate solution coincides with that produced by the bottom-up phase (Fig 3). Each of the subsequent steps processes exactly one C- or P-gap. At steps 1, 2, 4 and 5, rule R2.2 is employed and a different choice from the one depicted could have been made.

**Example for the top-down phase.** The top-down phase starting from the labeled gaps shown in Fig 3 produces 16 different optimal solutions. (Because there are four C-gaps and each C-gap can be processed in two possible ways by rule R2.2.) Fig 4 shows how the top-down phase constructs one of these optimal solutions.

At *x*, both C-gaps are reset as 0-gaps by one of the two choices for rule R2.2 (steps 1,2 in Fig 4), and the P-gap is set to 1 by rule R2.3, as *r* has no 0-gap at the interval [10,10] (step 3). At *y*, we only apply rule R2.2: C-gap [2,3] is replaced by 0-gap [2,2], which is a 0-gap at *x* (in practice this sets $A_{y}^{+}[2,3]$ as a copy of $A_{x}^{+}[2,3]$; see step 4 in Fig 4); the C-gap [5,7] at *y* is instead reset as a 0-gap (step 5).

**Fig 5:**
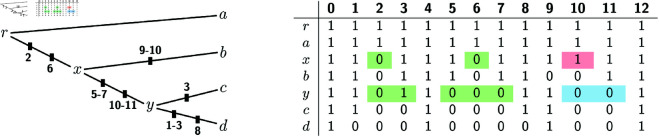
An optimal solution *A*^ + ^ for the example in Fig 1, found following the steps detailed in Fig 4. (Left:) Tree representation of *A*^ + ^, where each of the 8 deletions in *A*^ + ^ is depicted as a black box, as in Fig 2. (Right:) Table representation of *A*^ + ^. Colored rectangles indicate sites previously covered by labeled gaps.

The optimal solution produced in the end is depicted in its entirety in Fig 5.

## 4. Correctness of the algorithm

In order to prove that our algorithm finds all (and only) the optimal solutions of the DPP, we need the following key results.

**Proposition 1.** At the end of the bottom-up phase, site *k* is not in a labeled gap at *w* if and only if $A_{w}^{+}[k]=1$ for any candidate solution *A*^ + ^ of the DPP.

**Proposition 2.** If [i,j] is set as a 0-gap at *w* during the bottom-up phase then, in every optimal solution *A*^ + ^ of the DPP, $A_{w}^{+}[i,j]$ is a gap.

**Proposition 3.** If [i,j] is set as a P-gap at *w* in the bottom-up phase then, in every optimal solution of the DPP, $A_{w}^{+}[i,j]=A_{p}^{+}[i,j]$ with *p* being the parent of *w*.

**Proposition 4.** If [i,j] is set as a C-gap at *w* in the bottom-up phase then, in every optimal solution *A*^ + ^ of the DPP, $A_{w}^{+}[i,j]$ is a gap or $A_{w}^{+}[i,j]=A_{p}^{+}[i,j]$ with *p* being the parent of *w*.

The four propositions above, together with a few other auxiliary results, allow us to prove the correctness of the algorithm. All proofs can be found in S1 Text.

**Theorem 1.** *A*^ + ^ is among the alignment extensions constructed by Algorithm 1 if and only if *A*^ + ^ is an optimal solution of the DPP.

## 5. Graph-based representation of solutions

Since the top-down phase can generate a number of solutions exponential in the number of C-gaps, we now describe a way to derive a compact representation of the set of all DPP-optimal reconstructions for any given node. It consists of a collection of graphs, computable in polynomial time.

For each internal node w∈V(T)⧵L(T), we define a directed graph $G_{w}=(V_{w},E_{w})$, where $V_{w}=[0,m+1]$ is the same for every *w*, and coincides with the set of sites. As for the set of arcs *E*_*w*_, it is defined by the following algorithm, which should be executed after the bottom-up phase, and replaces the top-down phase of the algorithm described in Sect 3. We call this the *graph construction phase*.

The graph construction phase again visits the nodes of the tree with a pre-order traversal. Thus, for any non-root node *w* with parent *p*, by the time *w* is traversed *E*_*p*_ has already been constructed. Moreover the set *S*_*w*_ of labeled gaps at *w* is also available.

Initially set *E*_*w*_ as follows:

R3.0 $E_{w}=\{(k,k+1):k\in [0,m]$ and neither *k* nor *k* + 1 are in a labeled gap at *w*}

Then, for each labeled gap [i,j] at *w*:

R3.1 If [i,j] is a 0-gap, then add arc (i−1,j+1) to *E*_*w*_.
R3.2 If [i,j] is a P-gap, then for each arc $(h,k)\in E_{p}$ with h,k∈[i−1,j+1], add (h,k) to *E*_*w*_.
R3.3 If [i,j] is a C-gap, then first add arc (i−1,j+1) to *E*_*w*_; next, for each arc $(h,k)\in E_{p}$ with h,k∈[i−1,j+1], add (h,k) to *E*_*w*_.

In other words, if we only consider the set of arcs in *E*_*w*_ that are between two vertices in [i−1,j+1], which we denote with $E_{w}[[i-1,j+1]]$, we have:


$$E_{w}[[i-1,j+1]]=\left\{ \begin{array}{cc} \left\{(i-1,j+1)\right\} & \text{if [}i,j\text{] is a 0-gap,}\\ E_{p}[[i-1,j+1]] & \text{if [}i,j\text{] is a P-gap,}\\ E_{p}[[i-1,j+1]]\cup \{(i-1,j+1)\} & \text{if [}i,j\text{] is a C-gap.} \end{array}\right.$$

Note that since the root *r*(*T*) has no labeled gap (see Corollary 2 in S1 Text), the algorithm sets $E_{r(T)}=\{(k,k+1):k\in [0,m]\}$.

**Correctness of the graph construction phase.** Theorem 2 below states that each directed path in *G*_*w*_ from the first to the last site corresponds to one possible way of setting $A_{w}^{+}$ optimally, and inversely every optimal $A_{w}^{+}$ corresponds to one such path. Thus *G*_*w*_ provides a compact representation of all the optimal ancestral reconstructions for node *w*.

More formally, the graph *G*_*w*_ verifies the following property, where we recall that $𝟙(A_{w}^{+})=\{k\in [0,m+1]:A_{w}^{+}[k]=1\}$. The proof can be found in S1 Text (Sect 2).

**Theorem 2.** Let V⊆[0,m+1] and w∈V(T)⧵L(T).

*V* is the vertex set of a directed path from 0 to *m* + 1 in *G*_*w*_ if and only if $\exists A^{+}$ optimal solution of the DPP such that $V=𝟙(A_{w}^{+})$.

**Example for the graph construction phase.** Suppose we executed the bottom-up phase on the input of Fig 1, producing the labeled gaps in Fig 3. The graph construction phase starts by constructing *G*_*r*_, which (as noted above) is the trivial graph where two vertices are only adjacent if they are consecutive.

**Fig 6:**
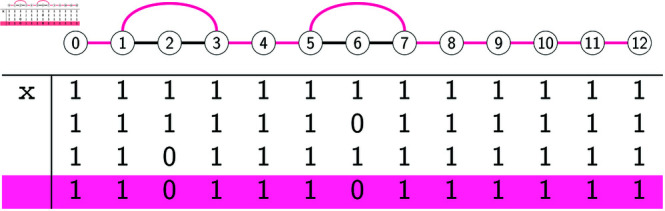
Graph representation of all possible $A_{x}^{+}$, where *A*^ + ^ is constrained to be an optimal solution for the example in Fig 1. (Top:) The graph *G*_*x*_ obtained by the graph construction phase, with one path highlighted. (Bottom:) The four possible $A_{x}^{+}$ when *A*^ + ^ is an optimal solution. By Theorem 2, there is a one-to-one correspondence between these $A_{x}^{+}$ and the paths from vertex 0 to vertex 12 in *G*_*x*_. The path highlighted in *G*_*x*_ corresponds to the highlighted $A_{x}^{+}$.

Next, $G_{x}=([0,12],E_{x})$ is set to the graph shown in Fig 6. In detail, rule R3.0 initially sets *E*_*x*_ = {(0, 1), (3, 4), (4, 5), (7, 8), (8, 9), (11, 12)} (as sites 2, 6 and 10 are in labeled gaps). Then rule R3.3 is applied to C-gap [2,2], which leads to adding first arc (1,3) and then arcs (1,2),(2,3) (copied from *G*_*r*_). Next, rule R3.3 is applied to C-gap [6,6], which similarly leads to adding arcs (5,7),(5,6),(6,7) to *E*_*x*_. Finally, rule R3.2 is applied to P-gap [10,10], which results in adding arcs (9,10),(10,11) (copied from *G*_*r*_). This completes the construction of *G*_*x*_.

**Fig 7:**
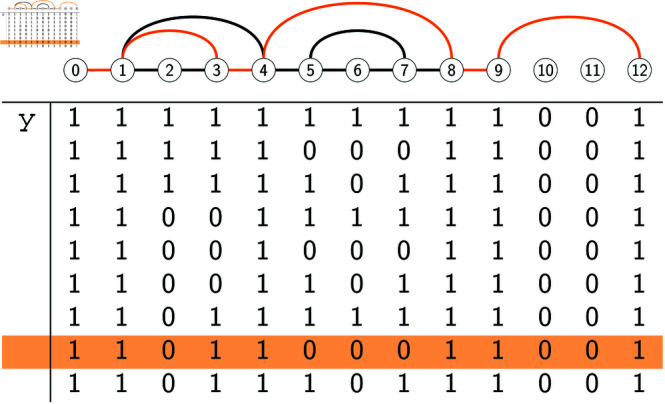
Graph representation of all possible $A_{y}^{+}$, where *A*^ + ^ is constrained to be an optimal solution for the example in Fig 1. (Top:) The graph *G*_*y*_ obtained by the graph construction phase, with one path highlighted. (Bottom:) The nine possible $A_{y}^{+}$ when *A*^ + ^ is an optimal solution. The path highlighted in *G*_*y*_ corresponds to the highlighted $A_{y}^{+}$.

Next, $G_{y}=([0,12],E_{y})$ is set to the graph shown in Fig 7. In detail, rule R3.0 initially sets *E*_*y*_ = {(0, 1), (8, 9)}, as the labeled gaps at *y* are [2,3],[5,7], [10,11]. Then rule R3.3 is applied to C-gap [2,3], which leads to adding to *E*_*y*_ arc (1,4) and then all the arcs in $E_{x}[[1,4]]$. Next, rule R3.3 is applied to C-gap [5,7], which leads to adding to *E*_*y*_ arc (4,8) and then all the arcs in $E_{x}[[4,8]]$. Finally, rule R3.1 is applied to 0-gap [10,11], which results in adding arc (9,12) to *E*_*y*_. This completes the construction of *G*_*y*_, and the graph construction phase terminates.

To illustrate Theorem 2, we focus on *G*_*y*_ (Fig 7). Theorem 2 implies that the set of paths in *G*_*y*_ from 0 to 12 is in a one-to-one correspondence with the set of binary strings that can be obtained as $A_{y}^{+}$ for some optimal solution *A*^ + ^.

To verify this, we can imagine deriving all 16 optimal solutions *A*^ + ^ and then taking the row $A_{y}^{+}$ in each of them. Doing so produces 9 different values for $A_{y}^{+}$, the binary rows depicted in Fig 7. (Sometimes pairs of optimal solutions that have different $A_{x}^{+}$ may have the same $A_{y}^{+}$, which explains why from 16 optimal solutions we only get 9 different $A_{y}^{+}$.) It is easy to check that each of these 9 values for $A_{y}^{+}$, when translated as $𝟙(A_{y}^{+})$, gives the vertex set of a path from 0 to 12 in *G*_*y*_. Conversely, each path in *G*_*y*_, when converted into the binary string that has a 1 at site *k* if the path traverses *k*, corresponds to one of the 9 rows shown in Fig 7.

## 6. Complexity

Here we state the main results about the complexity of our algorithms for the DPP. Full proofs can be found in S1 Text (Sect 3). The asymptotic complexity will be expressed as a function of the following parameters:

- *n*, the number of leaves of *T* or equivalently the number of rows of *A*;
- *s*, the number of optimal solutions of the DPP;
- *b*, the number of boundaries in *A*, where a *boundary* is defined as a pair of consecutive sites (k,k+1), such that A[k]≠A[k+1], i.e., their corresponding columns differ;
- *m*, the number of columns in *A* (excluding the first and last column which we only added for convenience of notation).

For the analysis of Algorithm 1, we assume that both the input alignment *A* and the output alignment *A*^ + ^ are encoded in *gap form*, instead of the table form presented in the Preliminaries (Sect 2.1). The gap form of an alignment *A* consists of encoding each row of *A* as the set of gaps it contains. For example, the input alignment of Fig 1 can be encoded as $A_{a}=\emptyset ,\;A_{b}=\{[2,2],[6,6],[9,10]\},\;A_{c}=\{[2,3],[5,7],[10,11]\},\;A_{d}=\{[1,3],[5,8],[10,11]\}$.

We assume that alignments are represented in gap form because, as a result, running times and memory requirements only depend on parameter *b* rather than *m*. In practice, for many alignments, we can expect *b* to be much smaller than *m*. We can now state the first of the two main results of this section.

**Theorem 3.** Assume that the input alignment *A* and every solution *A*^ + ^ in output is represented in gap form. Then Algorithm 1 runs in *O*(*nbs*) time.

Note that an obvious bound is $s\leq 2^{c}$, where *c* denotes the number of C-gaps at the end of the bottom-up phase. Although the worst-case complexity of Algorithm 1 is exponential in *c*, we can avoid the exponential factor in two ways. First, we can simply keep only one solution, which can be achieved by removing the duplication in rule R2.2 and skipping lines 7 – 9 in Algorithm 3. The complexity then becomes O(nb).

Alternatively the top-down phase can be replaced by the graph construction phase described in Sect 5. We now analyze the complexity of this algorithm. A key observation here is given by the following proposition.

**Proposition 5.** For any internal node *w*, *G*_*w*_ is a planar graph.

Proposition 5 can be used to obtain the other main complexity result:

**Theorem 4.** The algorithm that first runs the bottom-up phase and then the graph construction phase runs in O(nm) time.

Note that the complexity in Theorem 4 depends on *m* rather than on *b*. This is only because, for simplicity, we have chosen to describe the graph construction phase so that the graphs have [0,m+1] as vertex set. It is possible to see that it is feasible to modify the graph construction phase so that each graph has a vertex set of size O(b). Doing so would allow us to obtain an algorithm running in O(nb) time. We leave this as future work.

## 7. Practical relevance of the deletion-only case

Although this may not be evident at first sight, the relevance of deletion-only case extends beyond the solution of the DPP. In fact, algorithms to solve the DPP can be used to solve (parts of) many instances of the general problem (the IPP; Definition 5). This is due to three observations, which first we state informally and then illustrate with an example below.

1. Many instances of the IPP can be solved with a divide-and-conquer approach, by combining solutions for subproblems of the original problem. A way to do this was shown by [27], and another one is implicit in the work by [18].
2. The IPP is *time-reversible*, in the sense that if the input tree is rerooted on a node different from the original root, then every candidate solution retains its cost. As a consequence, optimal solutions of the IPP (excluding the reconstruction for the root) are independent of the position of the root.
3. Recall that phylogenetic correctness may constrain an internal node *x* to have many of its sites equal to 1. Now suppose that there exists a node *x* such that $A_{x}^{+}$ must only consist of 1s, for any candidate solution *A*^ + ^ of the IPP. Then if we re-root the tree in *x*, it is easy to see that none of the indels in any candidate solution *A*^ + ^ can be an insertion. But then we can solve the IPP by solving the DPP on the re-rooted tree with one of our algorithms.

**Fig 8:**
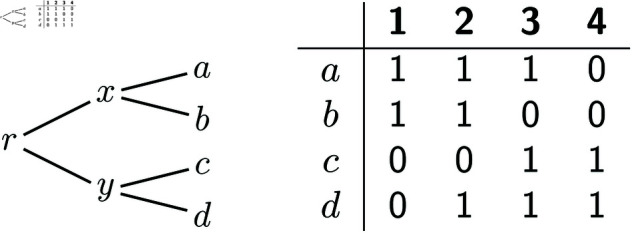
An instance (T,A) of the IPP that can be solved by applying the algorithms for the DPP.

The following example shows how these three ideas can be combined. Consider instance (T,A) of the IPP in Fig 8, where for simplicity we do not show the dummy columns *A*[0] and *A*[5]. While at first sight there is no reason why the deletion-only case would be relevant to solve this example, it turns out that we can indeed use our algorithms for the DPP to solve it.

First, this is a case where we can decompose the problem in two subproblems, i.e., an example of point 1 above. Namely, we can split the input alignment in two sub-alignments *A*_1_ and *A*_2_, where *A*_1_ consists of columns 1 and 2 of the original alignment, while *A*_2_ contains columns 3 and 4. The solutions for the instance in Fig 8 can be obtained by combining the solutions for the IPP on (*T*,*A*_1_) and those for the IPP on (*T*,*A*_2_). This can be proven by applying previous results [18,27], but the main idea here is that, as can be easily verified, every indel must be within one of the two sub-alignments *A*_1_ or *A*_2_.

Because (*T*, *A*_1_) and (*T*, *A*_2_) are symmetric, we can just concentrate on the instance (*T*, *A*_1_) of the IPP. Here phylogenetic correctness implies that every sequence on the path between *a* and *b* must have 1s at both sites 1 and 2. Thus, if we create a new node $r^{\prime }$ subdividing edge (x,a), we know that we must have $A_{1r^{\prime }}^{+}[1,2]=1\;1$. We then re-root the tree in $r^{\prime }$, which gives us a new rooted tree $T^{\prime }$ shown in Fig 8. For the reasons explained in points 2 and 3 above, solving the IPP on (*T*, *A*_1_) is equivalent to solving the DPP on $\left(T^{\prime },A_{1}\right)$. (Excluding the ancestral reconstruction for the root, which we discuss below.)

**Fig 9:**
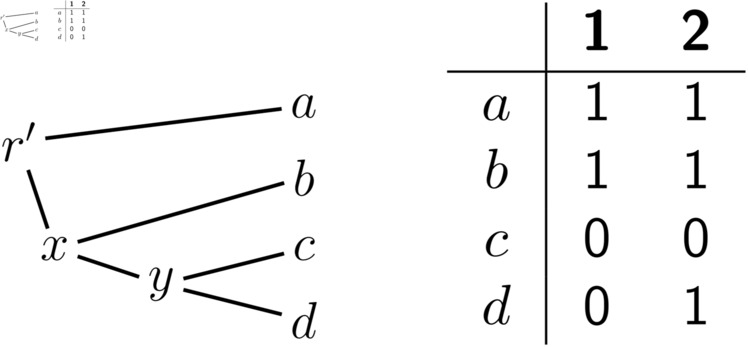
An instance $\left(T^{\prime },A_{1}\right)$ of the DPP that provides a solution of the first half of the IPP problem in Fig 8. This instance has two optimal solutions with $c(A_{1}^{+})=2$. Both solutions have $A_{1r^{\prime }}^{+}=A_{1x}^{+}=1\;1$, but they differ at node *y* where they have $A_{1y}^{+}\in \{1\;1\;,0\;1\}$.

It is now easy to check that, for the instance in Fig 9, our algorithm gives the two solutions with 1 1 at node *x* and with one of {11,01} at node *y*. By symmetry the IPP on (*T*, *A*_2_) has the two solutions with one of {11,10} at node *x* and with 1 1 at node *y*. Combining each solution for one sub-problem with each solution for the other sub-problem gives four solutions for the original IPP on (T,A) (Fig 8). They are the ones with $A_{x}^{+}\in \{1\;1\;1\;1\;,1\;1\;1\;0\}$ and $A_{y}^{+}\in \{1\;1\;1\;1\;,0\;1\;1\;1\}$. As for the root, it is easy to see that setting it equal to one of the reconstructions for its children always leads to an optimal solution. Thus, here we can set $A_{r}^{+}=A_{x}^{+}$ or $A_{r}^{+}=A_{y}^{+}$.

**Applicability of the DPP to a real-world dataset.** To investigate whether the strategy illustrated above can be applied frequently, we empirically evaluated it on OrthoMaM, a large collection of curated alignments of orthologous coding sequences [42]. Specifically, we downloaded OrthoMaM v12a [43], which includes 15,868 alignments from 190 complete mammalian genomes. Each alignment is available in four versions (nucleotide / amino acid, filtered / unfiltered) and is accompanied by a maximum-likelihood tree inferred from it. The filtered alignments are obtained by applying state-of-the-art alignment cleaning methods (implemented in MACSE, HMMcleaner and PhylteR; see [43] for details). For each alignment, both deletions and insertions are likely responsible for the gaps present in it.

We implemented the construction of the graph data structure introduced by [18]. In short, a consequence of this graph is that the set of gap characters in the input alignment (i.e. the set of pairs (x,k) for which $A_{x}[k]=0$) is partitioned into “components” so that the resolution of the IPP can be performed independently for each of the components. For each of the components, our code then tests if the tree can be re-rooted so that resolution of the IPP is equivalent to that of the DPP, as described in the example above. If this is the case, the component is classified as solvable, and not solvable otherwise. To facilitate interpretation of these results, we then classify alignment sites, rather than components, as not solvable, partially solvable or entirely solvable: a site *k* with gaps is *not solvable* if all of the gap characters at that site belong to components that are not solvable. A site *k* is *partially solvable* if it contains some gap characters belonging to a solvable component and some other gap characters belonging to a component that is not solvable. A site is *entirely solvable* if all of its gap characters belong to solvable components. The code to produce these statistics for any input alignment is available at https://gite.lirmm.fr/rivals/asr_indel.

Table 2 shows the results of the site classification procedure described above on the totality of the amino acid alignments, for the filtered and unfiltered OrthoMaM datasets. As shown under “gapped sites”, the filtering step results in the removal of about 30% of the sites with gaps across the entirety of the 15,868 alignments. In the filtered dataset, out of the sites with gaps about a quarter are entirely solvable, a quarter are partially solvable and half are not solvable at all. The proportion of sites with gaps that can be solved, partially or entirely, drops to about 30% for the unfiltered dataset.

**Table 2:** Experiment on the two OrthoMaM amino acid alignments datasets. For each dataset, we show the total number of sites with gaps (across 15,868 alignments), and the percentages of these sites that are entirely, partially, or not solvable by reduction to the DPP.

Dataset	gapped sites	entirely s.	partially s.	not solvable
*filtered*	8,439,079	26.80%	23.00%	50.20%
*unfiltered*	12,070,178	15.07%	15.78%	69.15%

Aside from showing that the DPP can be applied relatively frequently, the fact that the percentages of solvability drop for the unfiltered alignments shows that the parts of the alignments that are filtered out tend to pose more problems to our approach. This is not unexpected, as alignment cleaning methods tend to filter out the low-quality portions of multiple alignments, which are also the ones that are most likely to violate the assumptions necessary for the reduction to the DPP (presence of an internal node that can be used as a root, phylogenetic correctness, etc.).

## 8. Discussion

**Novel contributions and related works.** We have focused on a version of the Indel Parsimony Problem where only deletions are allowed (the DPP; Definition 6). We have described an exact algorithm that constructs all the optimal solutions for the DPP, and proved it is correct. While the algorithm’s running time may be exponential in the size of the input, this is only due to the exponential number of solutions. The algorithm can be easily changed to only find a single optimal solution in polynomial time (e.g. skip lines 7 – 9 in Algorithm 3). We note that an algorithm to find an optimal solution of the DPP was also proposed by [27]. Unfortunately, the pseudo-code provided in that paper appears to be incorrect, although the main underlying ideas are sound. See S1 Text, Sect 4, where we copied the pseudo-code and provide a simple example where it fails to return an optimal solution. Importantly, the main difference with our work is that we seek the whole set of optimal solutions, a goal which significantly complicates the task.

Two recently published parsimony-based works also deserve some discussion [25,26]. Iglhaut et al. [25] proposed an extension of Fitch’s algorithm [9] that handles insertions and deletions. The proposed algorithm is not intended to solve any explicit formulation of the parsimony principle for indels. Importantly, like in the original algorithm by Fitch, ancestral reconstruction is executed one site at a time, independently. Second, Tule et al. [26] present a Mixed-Integer Programming (MIP) formulation of indel inference, which provides an exact solution, albeit not polynomial in the worst case. However, their formulation is radically different from that of the Indel Parsimony Problem [27]. For example, phylogenetic correctness is not imposed, and the number of indel events between the reconstructions for two adjacent nodes can be very different (e.g. [26] count three separate indel events between 1 0 1 0 1 0 1 0 1 and 1 0 0 0 0 0 0 0 1 whereas Definition 4 only one).

Back to our paper, since there is no reason to favor one optimal scenario over an equally-parsimonious one, we consider it important to conceive ways to represent compactly the diversity of the reconstructed solutions. Our other main contribution is that we have shown that, in the context of the DPP, it is possible to efficiently construct a graph *G*_*w*_ representing the set of optimal reconstructions $A_{w}^{+}$ for any internal node *w* of the phylogeny. While the software GRASP does indeed construct similar graphs [23], we note that there is no proven global optimality guarantee for the reconstructions represented by the graphs of GRASP. We believe that our result (i.e. Theorem 2) is the first to provide a mathematical justification for the use of partial order graphs [40] in the context of ancestral sequence reconstruction. More recently, Tule et al. [26] also infer a graph for each internal node, but each of these graphs only has a single path from the first to the last site, meaning that it cannot be used to represent multiple reconstructions.

Naturally, we have derived the asymptotic complexity for all our algorithms. These algorithms are asymptotically optimal, because their running time is linear in the size of their inputs, or in the size on memory of its intended output (the set of all optimal solutions, or the graphs representing them).

Finally, we have shown that restricting the model to deletions is not as significant a limitation as it may initially seem. In an experiment on 15,868 alignments from a real-world dataset, we found that about 30% to 50% of the sites (for unfiltered and filtered alignments, respectively) can be at least partially solved by using our algorithms, even though the optimal solutions involve both deletions and insertions.

**Beyond unit costs and binary trees.** In both the IPP and the DPP (Definitions 5 and 6) each indel has a constant unit cost. However, it may be biologically relevant to consider more general indel cost functions. For example, it may be interesting to penalize longer indels by setting their cost with the affine function α+βℓ, where ℓ is the length of the indel [18,26,27]. Additionally the cost of insertions may be different from that of deletions (in the IPP), or the cost of an indel may depend on the edge where it occurs, allowing to model edge lengths [27]. These observations naturally raise the question of whether our algorithms can be extended to solve these more general formulations. While we leave the treatment of affine and asymmetric costs as open questions, it is interesting to note that the DPP with edge-dependent costs can also be solved using an extension of our techniques.

In S1 Text, Sect 5, we describe how our algorithms can be extended to solve the DPP where for each edge (u,v)∈E(T) we specify a cost $c_{uv}$ for the deletions occurring from *u* to v. The only thing that needs to be changed is the way labels 0, P and C are assigned to labeled gaps during the bottom-up phase. All other aspects of our algorithms remain unchanged, including the fact that we can represent all optimal solutions using the graph construction phase of Sect 5. To define the label of each labeled gap, we compute by dynamic programming two numerical scores describing the consequences of taking a deletion at that gap, and those of not taking it. Depending on the difference between these two scores, it may always be convenient to take a deletion at that labeled gap —in which case we assign label 0— never convenient to take a deletion —implying label P— or equally convenient —implying label C. See S1 Text for full details, including a pseudocode that replaces Algorithm 2.

We remark that this modified algorithm can also be used to solve the DPP on a non-binary tree *T*: to that end it suffices to take a binary resolution *T*^′^ of *T* and assign an infinite (or sufficiently large) cost to each edge of $T^{\prime }$ with no corresponding edge in *T*.

**Open problems.** The computational complexity result given by [27] implied the NP-hardness of two versions of the IPP:

- the IPP on trees with unbounded degree,
- the IPP on binary trees with branch-dependent indel costs.

This notably leaves open whether it is NP-hard to solve the IPP on binary trees and unit costs. In fact, we have chosen to tackle the DPP for binary trees and unit costs because of its potential impact on this open problem. While it may be interesting to clarify whether it is possible to extend our algorithms to more general indel costs —as discussed above— we believe that the most important avenue is to investigate whether our approach can be leveraged to also solve the general IPP on binary trees and unit costs. Should this prove impossible, it may be interesting to investigate more extensively its limitations on real-world data —for example with datasets covering a wider phylogenetic range than OrthoMaM— and to provide heuristics for the parts of the input alignments that cannot be solved exactly with our algorithms.

## Supporting information

S1 Text Results not fully detailed in the main text, including proofs of all mathematical statements. (PDF)

## References

[1] Hendrikse NM, Holmberg Larsson A, Svensson Gelius S, Kuprin S, Nordling E, Syrén P-O. Exploring the therapeutic potential of modern and ancestral phenylalanine/tyrosine ammonia-lyases as supplementary treatment of hereditary tyrosinemia. Sci Rep. 2020;10(1):1315. doi: 10.1038/s41598-020-57913-y 31992763 PMC6987202

[2] Ladics GS, Han K-H, Jang MS, Park H, Marshall V, Dersjant-Li Y, et al. Safety evaluation of a novel variant of consensus bacterial phytase. Toxicol Rep. 2020;7:844–51. doi: 10.1016/j.toxrep.2020.07.004 32714839 PMC7372152

[3] Ducatez MF, Bahl J, Griffin Y, Stigger-Rosser E, Franks J, Barman S, et al. Feasibility of reconstructed ancestral H5N1 influenza viruses for cross-clade protective vaccine development. Proc Natl Acad Sci U S A. 2011;108(1):349–54. doi: 10.1073/pnas.1012457108 21173241 PMC3017181

[4] Chang BSW, Jönsson K, Kazmi MA, Donoghue MJ, Sakmar TP. Recreating a functional ancestral archosaur visual pigment. Mol Biol Evol. 2002;19(9):1483–9. doi: 10.1093/oxfordjournals.molbev.a004211 12200476

[5] Blanchette M, Green ED, Miller W, Haussler D. Reconstructing large regions of an ancestral mammalian genome in silico. Genome Res. 2004;14(12):2412–23. doi: 10.1101/gr.2800104 15574820 PMC534665

[6] Redelings BD, Holmes I, Lunter G, Pupko T, Anisimova M. Insertions and deletions: computational methods, evolutionary dynamics, and biological applications. Mol Biol Evol. 2024;41(9):msae177. doi: 10.1093/molbev/msae177 39172750 PMC11385596

[7] Linard B, Swenson K, Pardi F. Rapid alignment-free phylogenetic identification of metagenomic sequences. Bioinformatics. 2019;35(18):3303–12. doi: 10.1093/bioinformatics/btz068 30698645

[8] Romashchenko N, Linard B, Pardi F, Rivals E. EPIK: precise and scalable evolutionary placement with informative k-mers. Bioinformatics. 2023;39(12):btad692. doi: 10.1093/bioinformatics/btad692 37975872 PMC10701097

[9] Fitch WM. Toward defining the course of evolution: minimum change for a specific tree topology. Systemat. Biol. 1971;20(4):406–16. doi: 10.1093/sysbio/20.4.406

[10] Sankoff D. Minimal mutation trees of sequences. SIAM J Appl Math. 1975;28(1):35–42. doi: 10.1137/0128004

[11] Yang Z, Kumar S, Nei M. A new method of inference of ancestral nucleotide and amino acid sequences. Genetics. 1995;141(4):1641–50. doi: 10.1093/genetics/141.4.1641 8601501 PMC1206894

[12] Koshi JM, Goldstein RA. Probabilistic reconstruction of ancestral protein sequences. J Mol Evol. 1996;42(2):313–20. doi: 10.1007/BF02198858 8919883

[13] Schluter D, Price T, Mooers AØ, Ludwig D. Likelihood of ancestor states in adaptive radiation. Evolution. 1997;51(6):1699–711. doi: 10.1111/j.1558-5646.1997.tb05095.x 28565128

[14] Pupko T, Pe’er I, Shamir R, Graur D. A fast algorithm for joint reconstruction of ancestral amino acid sequences. Mol Biol Evol. 2000;17(6):890–6. doi: 10.1093/oxfordjournals.molbev.a026369 10833195

[15] Snir S, Pachter L. Phylogenetic profiling of insertions and deletions in vertebrate genomes. In: Annual International Conference on Research in Computational Molecular Biology. 2006. p. 265–80.

[16] Diallo AB, Makarenkov V, Blanchette M. Exact and heuristic algorithms for the indel maximum likelihood problem. J Comput Biol. 2007;14(4):446–61. doi: 10.1089/cmb.2007.A006 17572023

[17] Snir S, Pachter L. Tracing the most parsimonious indel history. J Comput Biol. 2011;18(8):967–86. doi: 10.1089/cmb.2010.0325 21728862

[18] Fredslund J, Hein J, Scharling T. A large version of the small parsimony problem. In: Algorithms in Bioinformatics: Third International Workshop, WABI 2003, Budapest, Hungary, September 15–20, 2003. Proceedings 3. 2003. p. 417–32.

[19] Kim J, Sinha S. Indelign: a probabilistic framework for annotation of insertions and deletions in a multiple alignment. Bioinformatics. 2007;23(3):289–97. doi: 10.1093/bioinformatics/btl578 17110370

[20] Blanchette M, Diallo AB, Green ED, Miller W, Haussler D. Computational reconstruction of ancestral DNA sequences. Phylogenomics. 2008:171–84. 10.1007/978-1-59745-581-7_11 18629667

[21] Simmons MP, Ochoterena H. Gaps as characters in sequence-based phylogenetic analyses. Syst Biol. 2000;49(2):369–81. 12118412

[22] Ashkenazy H, Penn O, Doron-Faigenboim A, Cohen O, Cannarozzi G, Zomer O, et al. FastML: a web server for probabilistic reconstruction of ancestral sequences. Nucleic Acids Res. 2012;40(Web Server issue):W580-4. doi: 10.1093/nar/gks498 22661579 PMC3394241

[23] Foley G, Mora A, Ross CM, Bottoms S, Sützl L, et al. Engineering indel and substitution variants of diverse and ancient enzymes using Graphical Representation of Ancestral Sequence Predictions (GRASP). PLOS Comput Biol. 2022:18(10);e1010633. 10.1371/journal.pcbi.1010633 PMC9632902 36279274

[24] Jowkar G, Pečerska J, Maiolo M, Gil M, Anisimova M. ARPIP: ancestral sequence reconstruction with insertions and deletions under the poisson indel process. Syst Biol. 2023;72(2):307–18. doi: 10.1093/sysbio/syac050 35866991 PMC10275563

[25] Iglhaut C, Pečerska J, Gil M, Anisimova M. Please mind the gap: indel-aware parsimony for fast and accurate ancestral sequence reconstruction and multiple sequence alignment including long indels. Mol Biol Evol. 2024;41(7):msae109. doi: 10.1093/molbev/msae109 38842253 PMC11221656

[26] Tule S, Foley G, Zhao C, Forbes M, Bodén M. Optimal phylogenetic reconstruction of insertion and deletion events. Bioinformatics. 2024;40(Suppl 1):i277–86. doi: 10.1093/bioinformatics/btae254 38940131 PMC11211827

[27] Chindelevitch L, Li Z, Blais E, and Blanchette M. On the inference of parsimonious indel evolutionary scenarios. J Bioinform Comput Biol. 2006:4(3);721–44. 16960972 10.1142/s0219720006002168

[28] Diallo AB, Makarenkov V, Blanchette M. Finding maximum likelihood indel scenarios. In: Comparative Genomics: RECOMB 2006 International Workshop, RCG 2006 Montreal, Canada, September 24-26, 2006 Proceedings, 2006. p. 171–85.

[29] Diallo AB. Inference of insertion and deletion scenarios for ancestral genome reconstruction and phylogenetic analyses: algorithms and biological applications. 2009.

[30] Thorne JL, Kishino H, Felsenstein J. An evolutionary model for maximum likelihood alignment of DNA sequences. J Mol Evol. 1991;33(2):114–24. doi: 10.1007/BF02193625 1920447

[31] Thorne JL, Kishino H, Felsenstein J. Inching toward reality: an improved likelihood model of sequence evolution. J Mol Evol. 1992;34(1):3–16. doi: 10.1007/BF00163848 1556741

[32] Miklós I, Lunter GA, Holmes I. A “Long Indel” model for evolutionary sequence alignment. Mol Biol Evol. 2004;21(3):529–40. doi: 10.1093/molbev/msh043 14694074

[33] Bouchard-Côté A, Jordan MI. Evolutionary inference via the poisson indel process. Proc Natl Acad Sci U S A. 2013;110(4):1160–6. doi: 10.1073/pnas.1220450110 23275296 PMC3557041

[34] De Maio N. The cumulative indel model: fast and accurate statistical evolutionary alignment. Syst Biol. 2021;70(2):236–57. doi: 10.1093/sysbio/syaa050 32653921 PMC8559576

[35] Holmes IH. Historian: accurate reconstruction of ancestral sequences and evolutionary rates. Bioinformatics. 2017;33(8):1227–9. doi: 10.1093/bioinformatics/btw791 28104629 PMC6074814

[36] Westesson O, Barquist L, Holmes I. HandAlign: Bayesian multiple sequence alignment, phylogeny and ancestral reconstruction. Bioinformatics. 2012;28(8):1170–1. doi: 10.1093/bioinformatics/bts058 22285828 PMC3324510

[37] Zhang C, Huelsenbeck JP, Ronquist F. Using parsimony-guided tree proposals to accelerate convergence in bayesian phylogenetic inference. Syst Biol. 2020;69(5):1016–32. doi: 10.1093/sysbio/syaa002 31985810 PMC7440752

[38] Kramer AM, Thornlow B, Ye C, De Maio N, McBroome J, Hinrichs AS, et al. Online phylogenetics with matoptimize produces equivalent trees and is dramatically more efficient for large SARS-CoV-2 phylogenies than de novo and maximum-likelihood implementations. Syst Biol. 2023;72(5):1039–51. doi: 10.1093/sysbio/syad031 37232476 PMC10627557

[39] Felsenstein J. Evolutionary trees from DNA sequences: a maximum likelihood approach. J Mol Evol. 1981;17(6):368–76. doi: 10.1007/BF01734359 7288891

[40] Lee C, Grasso C, Sharlow MF. Multiple sequence alignment using partial order graphs. Bioinformatics. 2002;18(3):452–64. doi: 10.1093/bioinformatics/18.3.452 11934745

[41] Farris JS. Phylogenetic analysis under Dollo’s Law. Systemat Biol. 1977;26(1):77–88.

[42] Ranwez V, Delsuc F, Ranwez S, Belkhir K, Tilak M-K, Douzery EJ. OrthoMaM: a database of orthologous genomic markers for placental mammal phylogenetics. BMC Evol Biol. 2007;7:241. doi: 10.1186/1471-2148-7-241 18053139 PMC2249597

[43] Allio R, Delsuc F, Belkhir K, Douzery EJP, Ranwez V, Scornavacca C. OrthoMaM v12: a database of curated single-copy ortholog alignments and trees to study mammalian evolutionary genomics. Nucleic Acids Res. 2024;52(D1):D529–35. doi: 10.1093/nar/gkad834 37843103 PMC10767847

