# Supplementary material for: Algorithms to reconstruct past indels: The deletion-only parsimony problem
Source: PLoS Comput Biol. 2025 Jul 28;21(7):e1012585. doi: 10.1371/journal.pcbi.1012585 (PMC12331173; doi:10.1371/journal.pcbi.1012585)
Supplement: S1 Text — (PDF) [file pcbi.1012585.s001.pdf]

Supplementary Information to Algorithms to reconstruct past indels: the deletion-only parsimony problem.

This document contains all results not fully detailed in the main text, including proofs of all mathematical statements.

1 Correctness of the basic algorithm

All the propositions that follow are relative to a given instance (T, A) of the Deletion-only Parsimony problem (DPP).

Lemma 1. Let w be an internal node. If $[i, j]$ is a labeled gap at w at the end of the bottom-up phase, then all nodes descending from w must have a labeled gap that is a superset of $[i, j]$.

Proof. By induction. If w is a leaf, then it only has itself as descendant and the statement is trivially true. Otherwise, let u and v be the children of w. The bottom-up phase must have obtained $[i, j]$ as $[i_u, j_u] \cap [i_v, j_v]$ where $[i_u, j_u]$ is a labeled gap at u and $[i_v, j_v]$ is a labeled gap at v. By induction hypothesis, all nodes descending from u must have a labeled gap that is a superset of $[i_u, j_u] \supseteq [i, j]$, and similarly all nodes descending from v must have a labeled gap that is a superset of $[i_v, j_v] \supseteq [i, j]$. Thus the statement holds for w. □

Corollary 1. Let w be an internal node. If $[i, j]$ is a labeled gap at w at the end of the bottom-up phase, then all leaves descending from w must have a 0-gap that is a superset of $[i, j]$.

Proof. Trivial consequence of Lemma 1 and of the fact that every labeled gap at a leaf is a 0-gap. □

Corollary 2. The root $r(T)$ does not have any labeled gap.

Proof. If $[i, j]$ was a labeled gap at $r(T)$, then by Corollary 1 every leaf of T would have a 0-gap that is a superset of $[i, j]$. But then every site $k \in [i, j]$ would be such that $A_w[k] = 0, \forall w \in L(T)$, which violates the assumption that there are no columns only consisting of 0s (Sec. 2.1). □

Lemma 2. At the end of the bottom-up phase, site k is in a labeled gap at w if and only if $A_x[k] = 0, \forall x \in L(T_w)$.

Proof. If a site k is in a labeled gap at w, then $A_x[k] = 0, \forall x \in L(T_w)$ holds because of Corollary 1.

The opposite direction can be proven by induction on the height of w. If w is a leaf, $A_x[k] = 0, \forall x \in L(T_w)$ implies $A_w[k] = 0$, which in turn implies that site k is in a 0-gap at w. Now suppose that w has children u and v, and that $A_x[k] = 0, \forall x \in L(T_w)$. This implies $A_x[k] = 0, \forall x \in L(T_u)$ and $A_x[k] = 0, \forall x \in L(T_v)$. Then, by induction hypothesis, site k must be both in a labeled gap $[i_u, j_u]$ at u, and in a labeled gap $[i_v, j_v]$ at v. But then site k must also be in their intersection $[i, j] = [i_u, j_u] \cap [i_v, j_v]$, which is a labeled gap at w. □

Corollary 3. Let p be the parent node of w. At the end of the bottom-up phase, if a site k is not in a labeled gap at w, then k is also not in a labeled gap at p.

Proof. By Lemma 2, if k is not in a labeled gap at w, then $\exists x \in L(T_w) \subseteq L(T_p)$ such that $A_x[k] = 1$, meaning that k is not in a labeled gap at p. □

Lemma 3. Let w be a node. If w is a leaf, and $[i, j]$ is a labeled gap at w, then $[i, j]$ is a 0-gap at w. If w is an internal node, let u and v be its two children. At the end of the bottom-up phase, the following holds:

1. If $[i, j]$ is a 0-gap at w then there exist x_u leaf of T_u and x_v leaf of T_v, such that $[i, j]$ is a 0-gap at x_u and x_v.

2. If $[i, j]$ is a C-gap at w then exactly one of T_u and T_v has a leaf x such that $[i, j]$ is a 0-gap at x.

3. If $[i, j]$ is a P-gap at w then neither T_u nor T_v has a leaf x such that $[i, j]$ is a 0-gap at x.

Proof. If w is a leaf, the statement is trivially true. Now, suppose w is an internal node, and that the lemma is verified for u and v (induction hypothesis). Let $[i_u, j_u]$ and $[i_v, j_v]$ be the labeled gaps in u and v respectively, such that $[i, j] = [i_u, j_u] \cap [i_v, j_v]$.

If $[i, j]$ is a 0-gap at w then rule R1.1 must have been applied. But then $[i, j] = [i_u, j_u] = [i_v, j_v]$ and $[i, j]$ is not a P-gap at either u or v. The induction hypothesis applied to u implies that because $[i, j]$ is either a 0-gap or a C-gap at u, there exists at least one leaf in T_u with $[i, j]$ as a 0-gap. The same argument holds for v. Therefore, w has at least one leaf in each of its subtrees with $[i, j]$ as a 0-gap. This proves point 1 in the statement.

Second, if $[i, j]$ is a C-gap at w, then it has to be obtained via one of rules R1.2 or R1.4. If R1.2 is applied, then $[i, j] = [i_u, j_u] = [i_v, j_v]$ and without loss of generality, suppose that $[i, j]$ is a P-gap at u but not at v. The induction hypothesis applied to u and v implies that there is no leaf x_u in T_u with a 0-gap $[i, j]$, but there is at least one leaf x_v in T_v with a 0-gap $[i, j]$. Thus, w has such a leaf in exactly one of T_u, T_v. If R1.4 is applied, then without loss of generality, $[i, j] = [i_u, j_u] \subsetneq [i_v, j_v]$ and $[i, j]$ is not a P-gap at u. The induction hypothesis applied to u implies that there is a leaf x_u in T_u with $[i, j]$ as a 0-gap. Moreover, by Corollary 1, we know that any leaf x_v in T_v has $[i', j'] \supseteq [i_v, j_v] \supsetneq [i, j]$ as a 0-gap, meaning that $[i, j]$ cannot be a 0-gap at x_v. Thus, exactly one of T_u, T_v has a leaf having $[i, j]$ as a 0-gap. This proves point 2 in the statement.

Finally, if $[i, j]$ is a P-gap at w, then it can only have been obtained via one of rules R1.3, R1.5, or R1.6. If R1.3 is applied, then $[i, j] = [i_u, j_u] = [i_v, j_v]$ and $[i, j]$ is a P-gap at u and v. The induction hypothesis implies that no leaf x in T_u or T_v has $[i, j]$ as 0-gap. If R1.5 is applied, then, without loss of generality, $[i, j] = [i_u, j_u] \subsetneq [i_v, j_v]$ and $[i, j]$ is a P-gap at u. The induction hypothesis applied to u implies that there is no leaf x in T_u such that $[i, j]$ is a 0-gap at x. And by Corollary 1, there is also no such leaf in T_v. If R1.6 is applied, then, without loss of generality, $[i_u, j_u] = [i - k, j]$ and $[i_v, j_v] = [i, j + l]$ with $k, l > 0$. By Corollary 1, it means that for every leaf x in T_u, x has a 0-gap $[i', j'] \supseteq [i - k, j] \supsetneq [i, j]$. Thus, it is impossible that $[i, j]$ is a 0-gap at any leaf of T_u. The same argument holds for T_v. This proves point 3 in the statement. □

Recall that a candidate solution of the DPP is a phylogenetically correct alignment extension A^+ where only deletions occur. Optimal solutions are candidate solutions of minimum cost $c(A^+)$.

Proposition 1. At the end of the bottom-up phase, site k is not in a labeled gap at w if and only if $A^+_w[k] = 1$ for any candidate solution A^+ of the DPP.

Proof. First recall that site k is in a labeled gap at w if and only if $A_x[k] = 0, \forall x \in L(T_w)$ (Lemma 2).

Thus, if k is not in a labeled gap at w, then at least one of its descendant leaves x is such that $A_x[k] = 1$. But then any candidate solution A^+ must satisfy $A^+_w[k] = 1$, by Observation 1.2. This proves the forward implication in the statement.

To prove the other direction of the statement, assume $A^+_w[k] = 1$ for all candidate solutions of the DPP. By contradiction suppose that k is in a labeled gap at w. Then, because $A_x[k] = 0, \forall x \in L(T_w)$, we can construct a candidate solution A^+ with a deletion occurring on a edge ancestral to w and at an interval containing k. This implies that there exists a candidate solution with $A^+_w[k] = 0$, contradicting the initial assumption. □

Lemma 4 (”Homogeneity of labeled gaps”)**.** At the end of the bottom-up phase, if $[i, j]$ is a labeled gap at node w, then sites $i-1$ and $j+1$ are not in any labeled gap at w.

Proof. The statement is trivially true if w is a leaf.

Suppose now that w is an internal node and that the statement is true for u and v, the two children of w. Let $[i_u, j_u]$ and $[i_v, j_v]$ be the labeled gaps at u and v respectively, such that $[i, j] = [i_u, j_u] \cap [i_v, j_v]$. By induction hypothesis, we know that sites $i_u - 1$ and $j_u + 1$ are not in any labeled gap at u. The same goes for $i_v - 1$ and $j_v + 1$ at v.

Because every labeled gap at w is obtained as the intersection of one labeled gap at u and one labeled gap at v, none of $\{i_u - 1, i_v - 1, j_u + 1, j_v + 1\}$ can be in a labeled gap at w. Because $\{i - 1, j + 1\} \subseteq \{i_u - 1, i_v - 1, j_u + 1, j_v + 1\}$, the statement follows. □

Corollary 4. If $[i, j]$ is set as a labeled gap at node w in the bottom-up phase, then in every candidate solution A^+ of the DPP, $A^+_w[i - 1] = A^+_w[j + 1] = 1$.

Proof. Directly from Proposition 1 and Lemma 4. □

Lemma 5. Let A be an alignment for set $X \supseteq \{x, y\}$. Suppose changing A into A' so that $A'_x[i, j]$ and $A'_y[i, j]$ are now only composed of 0s, while leaving the rest of A_x and A_y unchanged. This change

1. removes all indels between x and y acting at an interval contained in $[i, j]$;
2. cannot increase the number of the other indels between x and y, i.e. those at an interval not contained in $[i, j]$.

Proof. Point 1 is trivially true. As for point 2, let us prove that the number of deletions at an interval not contained in $[i, j]$ cannot increase. The proof for insertions is symmetric.

Let K be the set of sites $k \in [1, i - 1] \cup [j + 1, m]$ such that $(A_x[k], A_y[k]) = (1, 0)$. Define partition $P(A)$ of K as the partition that puts two sites k_1, k_2 in

the same subset if and only if there is no site h with $k_1 < h < k_2$ such that $(A_x[h], A_y[h]) = (1,1)$. A different partition $P(A')$ of K is obtained in the same way for A' instead of A. Clearly $|P(A)|$ equals the number of deletions from A_x to A_y not contained in $[i,j]$, while $|P(A')|$ equals the number of deletions of from A'_x to A'_y not contained in $[i,j]$. Point 2 of the statement is then equivalent to proving $|P(A)| \geq |P(A')|$.

Now note that because A' may remove from A sites such that $(A_x[k], A_y[k]) = (1,1)$, then $P(A')$ must be a coarser partition than $P(A)$, meaning that each element of $P(A)$ is a subset of some element of $P(A')$. This implies $|P(A)| \geq |P(A')|$. □

The following Observation introduces a way of partitioning the set of deletions in any A^+ candidate solution of the DPP. We will use this partition in the proofs of several lemmas below. (Namely Propositions 2, 3, 4 and Lemma 6.)

Observation 2. Let A^+ be a candidate solution of the DPP. Suppose $[i,j]$ is set as a labeled gap at node w in the bottom-up phase. Let p be the parent node of w. Define the following sets of deletions:

- $D_{p,w}$ contains the deletions from p to w at a subinterval of $[i,j]$,
- D_{in} contains the deletions occurring in T_w at a subinterval of $[i,j]$,
- D_{out} contains the deletions occurring in T_w at an interval that is not contained in $[i,j]$,
- D_{else} contains the deletions occurring outside of $E(T_w) \cup \{(p,w)\}$ or in (p,w) at an interval with no common site with $[i,j]$.

Then, $\{D_{p,w}, D_{in}, D_{out}, D_{else}\}$ is a partition of the set of deletions in A^+.

Proof. Any deletion in A^+ occurs on some edge $(x,y) \in E(T)$ at some interval $[i',j']$. Depending on whether $[i',j'] \subseteq [i,j]$, or $[i',j'] \cap [i,j] = \emptyset$ or the remaining case, and on whether $(x,y) = (p,w)$, or $(x,y) \in E(T_w)$ or $(x,y) \notin E(T_w) \cup \{(p,w)\}$, we have 9 possible cases, depicted in Table S1. The four sets defined above correspond to a partition of these 9 cases. The only case that they do not cover (bottom-left case in Table S1) is not possible: Corollary 4 implies $A^+_w[i-1] = A^+_w[j+1] = 1$, meaning that any deletion in (p,w) is at an interval that is either contained in $[i,j]$ (i.e. the deletion is in $D_{p,w}$), or not overlapping with it (i.e. the deletion is in D_{else}). □

$[i',j']\dots$	(p,w)	$E(T_w)$	else
$\dots \subseteq [i,j]$	$D_{p,w}$	D_{in}	D_{else}
$\cap[i,j] = \emptyset$	D_{else}	D_{out}	D_{else}
else	$\emptyset$	D_{out}	D_{else}

Table S1: Partitioning deletions with respect to a labeled gap $[i,j]$ at w. Suppose a deletion occurs on edge (x,y) at interval $[i',j']$. The rows correspond to three different ways $[i',j']$ can be related to $[i,j]$. The columns correspond to the three cases $(x,y) = (p,w)$, $(x,y) \in E(T_w)$ and $(x,y) \notin E(T_w) \cup \{(p,w)\}$.

Proposition 2. If $[i,j]$ is set as a 0-gap at w during the bottom-up phase then, in every optimal solution A^+ of the DPP, $A^+_w[i,j]$ is a gap.

Proof. If w is a leaf, $A_w[i,j]$ must be a gap and therefore every extension A^+ of A must be such that $A_w^+[i,j]$ is a gap. Now suppose w is not a leaf. Since $[i,j]$ is set as a 0-gap for w in the bottom-up phase, it means by Lemma 3 that there exist $x_u \in L(T_u)$ and $x_v \in L(T_v)$, with u and v children of w, such that $[i,j]$ is a 0-gap at both x_u and x_v. And with Corollary 1, we know that every leaf x in T_w has a 0-gap $[i_x, j_x] \supseteq [i,j]$. We reason by contradiction and suppose there exists an optimal deletion-only solution A^+ where $[i,j]$ is not a gap for w. Let us partition the set of deletions in A^+ as described in Observation 2. We then have $c(A^+) = |D_{p,w}| + |D_{in}| + |D_{out}| + |D_{else}|$.

First note that $A_w^+[i-1] = A_w^+[j+1] = 1$, by Corollary 4. Since we assumed that $A_w^+[i,j]$ is not a gap, $\exists k \in [i,j]$ such that $A_w^+[k] = 1$. Note that at least one deletion at an interval including site k must occur in the the path from w to x_u. This deletion must belong to D_{in}, because if it acted on an interval not contained in $[i,j]$ but overlapping with $[i,j]$, then this would contradict the fact that $[i,j]$ is a 0-gap at x_u. We can reason similarly for x_v, and thus conclude $|D_{in}| \geq 2$.

Now, let us take B^+, a copy of A^+, and modify B^+ by setting $B_x^+[h] = 0$, $\forall x$ descendant of w and $\forall h \in [i,j]$. By doing so, we create a new deletion in B^+ between w and its parent p, at an interval $[i_0, j_0] \subseteq [i,j]$ containing all sites $k \in [i,j]$ such that $A_w^+[k] = 1$. Note that $i_0 = \min\{k \in [i,j] : A_p^+[k] = 1\}$ and $j_0 = \max\{k \in [i,j] : A_p^+[k] = 1\}$. B^+ is clearly an extension of A that only involves deletions, so it is a candidate solution of the DPP. Let $\{D'_{p,w}, D'_{in}, D'_{out}, D'_{else}\}$ be the partition of the deletions in B^+ defined in the same way as $\{D_{p,w}, D_{in}, D_{out}, D_{else}\}$ for A^+.

Now note that all deletions in D_{in} are no longer deletions in B^+, by Lemma 5. The same holds for the deletions in $D_{p,w}$, which in A^+ act on intervals that are strictly contained in the interval $[i_0, j_0]$ of the new deletion from p to w in B^+. Thus, $|D'_{in}| = 0$ and $|D'_{p,w}| = 1$.

As for D_{out}, Lemma 5 implies that the number of deletions in T_w at an interval not contained in $[i,j]$ cannot increase when switching from A^+ to B^+, meaning that $|D_{out}| \geq |D'_{out}|$.

Finally, we focus on D_{else}. First, it is easy to see that any deletion occurring outside $\{(p,w)\} \cup E(T_w)$ is unaffected by the changes between A^+ and B^+. Similarly, any deletion from p to w at an interval not overlapping with $[i,j]$ is unaffected by these changes because of $A_w^+[i-1] = A_w^+[j+1] = 1 = B_w^+[i-1] = B_w^+[j+1]$. Thus, $|D_{else}| = |D'_{else}|$.

In conclusion, $c(A^+) \geq 2 + |D_{p,w}| + |D_{out}| + |D_{else}| > 0 + 1 + |D'_{out}| + |D'_{else}| = c(B^+)$, which contradicts the assumption that A^+ is optimal. $\square$

Proposition 3. *If $[i,j]$ is set as a P-gap at w in the bottom-up phase then, in every optimal solution of the DPP, $A_w^+[i,j] = A_p^+[i,j]$ with p being the parent of w.*

Proof. We reason by contradiction and suppose there exists an optimal solution A^+ where $A_w^+[i,j] \neq A_p^+[i,j]$. We partition the set of deletions in A^+ as described in Observation 2. We then have $c(A^+) = |D_{p,w}| + |D_{in}| + |D_{out}| + |D_{else}|$. $A_w^+[i,j] \neq A_p^+[i,j]$ and $A_w^+[i-1] = A_w^+[j+1] = 1$ (consequence of Corollary 4), together imply $|D_{p,w}| \geq 1$.

Now set B^+ as a copy of A^+, and then $\forall x$ descendant of w, modify $B_x^+[i,j]$ as follows:

- if $A^+_x[i-1] = A^+_x[j+1] = 1$ then set $B^+_x[i,j] = A^+_p[i,j]$,
- otherwise set $B^+_x[h] = 0$, $\forall h \in [i,j]$.

Note that we necessarily set $B^+_w[i,j] = A^+_p[i,j]$, since $A^+_w[i-1] = A^+_w[j+1] = 1$ by Corollary 4. Moreover, by Lemma 3 the condition $A^+_x[i-1] = A^+_x[j+1] = 1$ cannot be true for any leaf in T_w. Thus, in any path between w and one of the leaves in T_w, there exists an edge (x,y) where that condition is true for x but false for y, meaning that we set $B^+_x[i,j] = A^+_p[i,j]$ and $B^+_y[i,j] = 0\dots0$. Let E be the set of such edges (x,y). Because for any edge $(x,y) \in E$ at least one of $A^+_y[i-1]$ or $A^+_y[j+1]$ equals 0, in A^+ there exists a deletion from x to y at an interval containing at least one of $i-1$ or $j+1$. This deletion is necessarily an element of D_{out}. Moreover, B^+ is an extension of A and only involves deletions, so it is a candidate solution of the DPP.

Let $\{D'_{p,w}, D'_{in}, D'_{out}, D'_{else}\}$ be the partition of the deletions in B^+ defined in the same way as $\{D_{p,w}, D_{in}, D_{out}, D_{else}\}$ for A^+. Note that $B^+_w[i,j] = A^+_p[i,j] = B^+_p[i,j]$ implies $|D'_{p,w}| = 0$. Moreover, for the same reasons as in the proof of Proposition 2, $|D_{else}| = |D'_{else}|$.

We now show that $|D_{out}| \geq |D'_{out}|$ by showing that for any $(x,y) \in E(T_w)$ there are at least as many deletions from x to y in D_{out} than in D'_{out}. We consider three cases, depending on whether $(x,y) \in E$, or (x,y) is a strict descendant of an element of E, or a strict ancestor. (We say that an edge (x,y) is a strict descendant of another edge (x',y') if x and y are strict descendants of x' and y', respectively. Strict ancestors are defined symmetrically.)

First, if (x,y) is a strict descendant of an element of E, then $B^+_x[i,j] = B^+_y[i,j] = 0\dots0$ and, by Lemma 5, the number of deletions from x to y cannot be larger in D'_{out} than in D_{out}.

Second, if $(x,y) \in E(T_w)$ is a strict ancestor of an element of E, then by construction all of $A^+_x[i-1], A^+_y[i-1], A^+_x[j+1], A^+_y[j+1]$ are equal to 1. But then, any deletion in D_{out} and D'_{out} from x to y must be at an interval that does not overlap with $[i-1, j+1]$. This means that the deletions from x to y are the same in D_{out} and D'_{out}, and in particular that their number does not change.

Third, if $(x,y) \in E$, as we noted earlier this means that a deletion from x to y in D_{out} occurs at an interval $[i',j']$ containing $i-1$ or $j+1$ (potentially both). But then, because of $B^+_y[i,j] = 0\dots0$, this deletion is replaced in B^+ by a potentially different deletion from x to y at an interval including $[i',j']$ and all sites $k \in [i,j]$ such that $B^+_x[k] = 1$. Clearly, this new deletion is an element of D'_{out}. Furthermore, any other deletion from x to y in D'_{out} was already present at the same interval in D_{out}. Thus, for every deletion from x to y in D'_{out} there exists at least one deletion in D_{out} that is either identical to it, or occurring on a smaller interval, which implies that there are at least as many deletions from x to y in D_{out} than in D'_{out}. This completes the proof of $|D_{out}| \geq |D'_{out}|$.

Finally, by considering the same three cases as we did to prove $|D_{out}| \geq |D'_{out}|$, it is easy to see that $|D'_{in}| = 0$. In conclusion, $c(B^+) = 0 + 0 + |D'_{out}| + |D'_{else}| < 1 + |D_{in}| + |D_{out}| + |D_{else}| \leq c(A^+)$, which contradicts the optimality of A^+. □

Proposition 4. If $[i,j]$ is set as a C-gap at w in the bottom-up phase then, in every optimal solution A^+ of the DPP, $A^+_w[i,j]$ is a gap or $A^+_w[i,j] = A^+_p[i,j]$ with p being the parent of w.

Proof. Since $[i, j]$ is set as a C-gap for w in the bottom-up phase, by Lemma 3 one child of w, say u, has a decendant leaf x_u such that $[i, j]$ is a gap at x_u, while the other child v is such that all its descendant leaves have a gap that is a proper superset of $[i, j]$. We reason by contradiction and suppose there exists an optimal solution A^+ of DPP where $A^+_w[i, j]$ is not a gap and $A^+_w[i, j] \neq A^+_p[i, j]$. Let us partition the set of deletions in A^+ as described in Observation 2. We then have $c(A^+) = |D_{p,w}| + |D_{in}| + |D_{out}| + |D_{else}|$.

First note that $A^+_w[i-1] = A^+_w[j+1] = 1$, by Corollary 4. Since we assumed that $A^+_w[i, j]$ is not a gap, $\exists k \in [i, j]$ such that $A^+_w[k] = 1$. Note that at least one deletion at an interval including site k must occur in the the path from w to x_u. This deletion must belong to D_{in}, because if it acted on an interval not contained in $[i, j]$ but overlapping with $[i, j]$, then this would contradict the fact that $[i, j]$ is a 0-gap at x_u. Thus, $|D_{in}| \geq 1$.

Moreover, the assumption that $A^+_w[i, j] \neq A^+_p[i, j]$ implies that there exists $k' \in [i, j]$ such that $A^+_p[k'] = 1$ and $A^+_w[k'] = 0$. Now note that $A^+_w[i-1] = A^+_w[j+1] = 1$ implies that the deletion from p to w that deletes site k' must occur at a subinterval of $[i, j]$. Therefore, $|D_{p,w}| \geq 1$.

Now, define the candidate solution B^+ in the same way as in the proof of Proposition 2, that is, $B^+_x[h] = 0$, $\forall x$ descendant of w and $\forall h \in [i, j]$, while B^+ is a copy of A^+ elsewhere. Let $\{D'_{p,w}, D'_{in}, D'_{out}, D'_{else}\}$ be the partition of the deletions in B^+ defined in the same way as $\{D_{p,w}, D_{in}, D_{out}, D_{else}\}$ for A^+. For the same reasons as those detailed in the proof of Proposition 2, we have $|D'_{in}| = 0$, $|D'_{p,w}| = 1$, $|D_{out}| \geq |D'_{out}|$ and $|D_{else}| = |D'_{else}|$.

Thus, $c(A^+) \geq 1 + 1 + |D_{out}| + |D_{else}| > 0 + 1 + |D'_{out}| + |D'_{else}| = c(B^+)$, which contradicts the assumption that A^+ is optimal.

□

Lemma 6. Let $w \in V(T) \setminus \{r(T)\}$ and let p be the parent of w. If $[i, j]$ is set as a C-gap at w in the bottom-up phase, then:

1. $\forall A^+$ optimal solution of DPP, such that $A^+_w[i, j]$ is a gap, $\exists B^+$ optimal solution of DPP, such that
   - $B^+_w[i, j] = B^+_p[i, j]$ and
   - $B^+_x[k] = A^+_x[k]$, whenever $x \in V(T) \setminus V(T_w)$ or $k \notin [i, j]$.
2. $\forall B^+$ optimal solution of DPP, such that $B^+_w[i, j] = B^+_p[i, j]$, $\exists A^+$ optimal solution of DPP, such that
   - $A^+_w[i, j]$ is a gap and
   - $A^+_x[k] = B^+_x[k]$, whenever $x \in V(T) \setminus V(T_w)$ or $k \notin [i, j]$.

Proof. Without loss of generality, suppose u is the child of w that has a descendant leaf x_u such that $[i, j]$ is a gap at x_u, while the other child v is such that all its descendant leaves have a gap that is a proper superset of $[i, j]$.

(*Proof of 1.*) Let A^+ be an optimal solution of DPP, such that $A^+_w[i, j]$ is a gap. If $A^+_p[i, j]$ is a gap, then we already have $A^+_w[i, j] = A^+_p[i, j]$, meaning that statement 1 is trivially true for $B^+ = A^+$. Now, suppose that $A^+_p[i, j]$ is not a gap. Let K be the non-empty set of sites $k \in [i, j]$ such that $A^+_p[k] = 1$. Let us partition the set of deletions in A^+ as described in Observation 2. Thus, we have $c(A^+) = |D_{p,w}| + |D_{in}| + |D_{out}| + |D_{else}|$, with $|D_{p,w}| = 1$, $|D_{in}| = 0$. ($|D_{p,w}| = 1$

is because a deletion from p to w occurs at $[\min K, \max K]$; $|D_{in}| = 0$ because $A^+_x[h] = 0$, $\forall x \in V(T_w), \forall h \in [i,j]$.)

Let us take B^+, a copy of A^+, and modify B^+ as follows: $\forall k \in K$ and $\forall x \in V(T_v) \cup \{w\}$, set $B^+_x[k] = 1$ if $A^+_x[i-1] = A^+_x[j+1] = 1$. This has the effect of setting $B^+_x[i,j] = B^+_p[i,j]$ $(= A^+_p[i,j])$ for $x = w$ and possibly for several descendants of v. Up until the end of this proof, let $\{D'_{p,w}, D'_{in}, D'_{out}, D'_{else}\}$ be the partition of the deletions in B^+ defined in the same way as $\{D_{p,w}, D_{in}, D_{out}, D_{else}\}$ for A^+. Clearly, $B^+_w[i,j] = B^+_p[i,j]$ implies $|D'_{p,w}| = 0$. Also note that $|D'_{in}| = 1$ because we created a deletion from w to u at interval $[\min K, \max K]$. Moreover, $|D'_{else}| = |D_{else}|$, because these deletions remain untouched.

Now let us focus on the "new" deletions in B^+, i.e. those in B^+ but not in A^+. Let (x,y) be an edge where a new deletion occurs. It is easy to see that the only way for a new deletion to arise in B^+ at edge (x,y) is that we have changed the sites in set K from 0s in A^+_x into 1s in B^+_x, but that these sites have remained 0s in B^+_y. This occurs for $(x,y) = (w,u)$, where the new deletion is the reason why $|D'_{in}| = 1$. The only other case when this occurs is for $(x,y) \in E(T_v) \cup \{(w,v)\}$ whenever $A^+_x[i-1] = A^+_x[j+1] = 1$ and $(A^+_y[i-1], A^+_y[j+1]) \neq (1,1)$. This means that an "old" deletion from x to y in D_{out} occurred at an interval $[i',j']$ containing $i-1$ or $j+1$ (potentially both). But then, it is easy to see that the new deletion in B^+ is identical to the old one, except that it occurs at the potentially larger interval $[\min(K \cup \{i'\}), \max(K \cup \{j'\})]$. This new deletion is clearly an element of D'_{out}.

In conclusion, aside from the new deletion in D'_{in}, any other new deletion in B^+ is an element of D'_{out} that replaces an element of D_{out} by extending its interval. This proves that $c(B^+) = 0 + 1 + |D'_{out}| + |D'_{else}| \leq |D_{p,w}| + |D_{in}| + |D_{out}| + |D_{else}| = c(A^+)$. Thus, B^+ is an optimal solution of the DPP, such that $B^+_w[i,j] = B^+_p[i,j]$.

(*Proof of 2.*) Let B^+ be an optimal solution of DPP, such that $B^+_w[i,j] = B^+_p[i,j]$. If $B^+_p[i,j]$ is a gap, then since $B^+_w[i-1] = B^+_w[j+1] = 1$ (Corollary 4) $B^+_w[i,j]$ is also a gap. Thus statement 2 is trivially true for $A^+ = B^+$. Now, suppose that $B^+_p[i,j]$ is not a gap. Thus, we have $c(B^+) = |D'_{p,w}| + |D'_{in}| + |D'_{out}| + |D'_{else}|$, with $|D'_{p,w}| = 0$ and $|D'_{in}| \geq 1$. Let us take A^+, a copy of B^+, and modify A^+ by setting $A^+_x[h] = 0, \forall h \in [i,j]$ and $\forall x \in V(T_w)$.

For the same reasons detailed in the proof of Proposition 2 we have $|D_{p,w}| = 1$, $|D_{in}| = 0$, $D'_{else} = D_{else}$ and, by Lemma 5, $|D'_{out}| \geq |D_{out}|$. Therefore, we have $c(A^+) \leq c(B^+)$. Thus A^+ is an optimal solution of DPP, such that $A^+_w[i,j]$ is a gap.

Finally note that both transformations described above ensure $A^+_x[k] = B^+_x[k]$ for $x \notin V(T_w)$ or $k \notin [i,j]$, thus completing the proof of the statement. □

In the following statement and in the proof of Theorem 2 we use the following notation.

Definition 7 (*concatenation*)**.** Let A and B be two alignments over X, let $x \in X$ and $k \in [0,m]$. $A_x[0,k]B_x[k+1,m+1]$ denotes the array of $(m+2)$ characters obtained by concatenating $A_x[0,k]$ and $B_x[k+1,m+1]$.

Lemma 7. Suppose that site k does not belong to any labeled gap at w, at the end of the bottom-up phase. For any two optimal solutions of the DPP D^+ and

E^+, there exists an optimal solution of the DPP F^+ such that

$$F_w^+ = D_w^+[0,k]\, E_w^+[k+1,m+1]. \tag{S1}$$

Proof. By induction on the depth of w. If $w = r(T)$, then D_w^+ and E_w^+ are only composed of 1s by Observation 1 and the statement trivially holds.

Now suppose $w \neq r(T)$ and that the statement holds for p, the parent of w. That is, there exists an optimal F^+ such that

$$F_p^+ = D_p^+[0,k]\, E_p^+[k+1,m+1]. \tag{S2}$$

F_w^+ is not guaranteed to satisfy Equation (S1), because of the possible presence of C-gaps at w (as we show below). We will see, however, that F^+ guarantees the existence of another optimal solution that does satisfy Equation (S1).

First note that for every site h outside labeled gaps at w, $F_w^+[h] = D_w^+[h] = E_w^+[h] = 1$ by Proposition 1, meaning that $F_w^+[h]$ is consistent with Equation (S1). Similarly, for every 0-gap $[i,j]$ at w, $F_w^+[i,j] = D_w^+[i,j] = E_w^+[i,j] = 0\ldots0$, by Proposition 2, meaning that $F_w^+[i,j]$ is consistent with Equation (S1).

Moreover, if $[i,j]$ is a P-gap at w with $i,j < k$ then $F_w^+[i,j] = F_p^+[i,j]$, $D_w^+[i,j] = D_p^+[i,j]$ (Proposition 3) and $F_p^+[i,j] = D_p^+[i,j]$ (Equation (S2)) imply $F_w^+[i,j] = D_w^+[i,j]$. Symmetrically, $F_w^+[i,j] = E_w^+[i,j]$ for every P-gap $[i,j]$ at w with $i,j > k$. So, whenever $[i,j]$ is a P-gap, $F_w^+[i,j]$ is consistent with Equation (S1).

The arguments above show that, if there are no C-gaps at w, then F_w^+ satisfies Equation (S1). Now consider a C-gap $[i,j]$ at w, and suppose, without loss of generality, $i,j < k$. By Proposition 4 we know that $F_w^+[i,j] \in \{0\ldots0, F_p^+[i,j]\}$ and $D_w^+[i,j] \in \{0\ldots0, D_p^+[i,j]\}$, with $F_p^+[i,j] = D_p^+[i,j]$ (Equation (S2)). If $F_w^+[i,j] \neq D_w^+[i,j]$, Lemma 6 guarantees the existence of another optimal solution G^+ such that $G_w^+[i,j] = D_w^+[i,j]$. Moreover G_w^+ only differs from F_w^+ at the sites in $[i,j]$.

Now note that G_w^+ may still not satisfy Equation (S1) because of the presence of other C-gaps at w. In this case, repeated application of Lemma 6 in the way we described above leads to prove the existence of an optimal solution H^+ such that (a) $H_w^+[i,j] = D_w^+[i,j]$ for every C-gap $[i,j]$ with $i,j < k$, (b) $H_w^+[i,j] = E_w^+[i,j]$ for every C-gap $[i,j]$ with $i,j > k$, (c) H_w^+ coincides with F_w^+ for every site that is not in a C-gap. Therefore H_w^+ satisfies Equation (S1). □

Theorem 1. *A^+ is among the alignment extensions constructed by Algorithm 1 if and only if A^+ is an optimal solution of the DPP.*

Proof. Recall that Algorithm 1 stores labeled gaps in a data structure S, which allows retrieving the set S_u of labeled gaps at u, $\forall u \in V(T)$. At any point during execution, multiple versions of S are stored in a set Sol. Each S represents an "intermediate solution", in the following sense. Let the "intermediate alignment extension" $I^+(S)$ be defined as follows:

- $I_w^+[k](S) = 0$ if $\exists$ a 0-gap $[i,j] \in S_w$ with $k \in [i,j]$;
- $I_w^+[k](S) = 1$ if, $\forall [i,j] \in S_w, k \notin [i,j]$;
- $I_w^+[k](S) = \mathit{undefined}$, if $\exists$ a C-gap or a P-gap $[i,j] \in S_w$ with $k \in [i,j]$.

In other words, $I^+(S)$ contains a 0 for every site that is in a 0-gap in S, a 1 for every site that is outside a labeled gap in S, and is undefined elsewhere. (Strictly speaking, $I^+(S)$ is not an alignment extension, because of the presence of undefined entries in it.) In the following we say that the intermediate solution S *is extendable to* alignment extension A^+ if we can set all the undefined entries in $I^+(S)$ so as to make it equal to A^+ (or, equivalently, if $I^+(S) = A^+$ for all entries where $I^+(S)$ is defined).

With the definitions above in mind, it is possible to prove the correctness of Algorithm 1 by proving the following two invariant properties. As we will show, they hold at any point during the execution of Algorithm 1 after Sol has been initialized.

Invariant 1 For any $S \in Sol$, S is extendable to an optimal solution.

Invariant 2 For any optimal solution A^+, there exists $S \in Sol$ extendable to A^+.

Before proving these two invariant properties, we note that they imply the Theorem's statement. This is because they must hold in particular at the very end of the execution of Algorithm 1. At this point, every $S \in Sol$ is such that all of the labeled gaps stored in it are 0-gaps, meaning that $I^+(S)$ has no undefined entries, and thus every $S \in Sol$ is only extendable to a single alignment extension $I^+(S)$. Thus Invariant 1 proves that, at termination, any $S \in Sol$ is such that $I^+(S)$ is an optimal solution. In other words, any returned solution is optimal. Invariant 2 proves that for any optimal solution A^+, there exists $S \in Sol$ such that $I^+(S) = A^+$. In other words, any optimal solution is among those returned.

Let us now prove the two invariant properties. First note that when Sol is initialized with $Sol \leftarrow \{S\}$, Proposition 1 says that the sites that are set to 1 in $I^+(S)$ are precisely those that equal 1 in all candidate solutions. Moreover by Proposition 2, all sites that are set to 0 in $I^+(S)$ are also equal to 0 in any optimal solution. These two observations mean that, initially, the only S in Sol is extendable to any optimal solution, which implies that both Invariant 1 and Invariant 2 are true at this stage.

As for later stages during execution, note that Sol can only be changed because of application of rules R2.1, R2.2, R2.3. Specifically, rules R2.1 and R2.3 replace an intermediate solution S_0 with another intermediate solution S'_0, while rule R2.2 replaces a intermediate solution S_0 with two intermediate solutions which we call S_1 and S_2 in this proof. To prove that the two invariant properties remain true after application of these rules, it suffices to prove that for any optimal solution A^+: (a) in rules R2.1 and R2.3, if S_0 is extendable to A^+, then S'_0 is also extendable to A^+; (b) in rule R2.2, if S_0 is extendable to A^+, then either S_1 or S_2 remains extendable to solution A^+, while the other is extendable to another optimal solution B^+.

Before considering each of rules R2.1, R2.2, R2.3, recall that they are applied to a labeled gap $[i, j]$ at a node w. Since the root $r(T)$ cannot have any labeled gaps (Corollary 2), we can assume $w \neq r(T)$. We call p the parent of w. Note that, by the time one of these rules is applied to a labeled gap at w, all of the C- and P-gaps at p have already been resolved, meaning that $I^+_p(S_0)$ does not contain any undefined site.

First, suppose rule R2.1 was applied, turning the C-gap $[i, j]$ at node w into a 0-gap. This replaces intermediate solution S_0 with S'_0, so that while $I^+_w[i, j](S_0)$ was a stretch of undefined sites, $I^+_w[i, j](S'_0)$ is now a gap. Now suppose S_0 is extendable to optimal solution A^+. Rule R2.1 presupposes that $I^+_p[i, j](S_0)$ is a gap, which implies that also $A^+_p[i, j]$ is a gap. But then Proposition 4 implies that $A^+_w[i, j]$ is also a gap. Because $I^+_w[i, j](S'_0)$ and $A^+_w[i, j]$ are both gaps, and $I^+_w[i, j](S'_0)$ is all that changed in $I^+(S'_0)$ with respect to $I^+(S_0)$, S'_0 is also extendable to A^+, which proves point (a) above for rule R2.1.

Second, suppose rule R2.3 was applied to intermediate solution S_0, replacing it with S'_0. In terms of $I^+(S)$, while $I^+_w[i, j](S_0)$ was a stretch of undefined sites, rule R2.3 has the effect of setting $I^+_w[i, j](S'_0) = I^+_p[i, j](S_0)$. Now suppose S_0 is extendable to optimal solution A^+, which implies that $I^+_p[i, j](S_0) = A^+_p[i, j]$. Proposition 3 implies $A^+_w[i, j] = A^+_p[i, j]$, which together with $I^+_w[i, j](S'_0) = I^+_p[i, j](S_0) = A^+_p[i, j]$ proven above implies $A^+_w[i, j] = I^+_w[i, j](S'_0)$. This means that S'_0 is also extendable to A^+, which proves point (a) above for rule R2.3.

Finally, suppose rule R2.2 was applied to intermediate solution S_0, replacing it in *Sol* with S_1 and S_2. While $I^+_w[i, j](S_0)$ was a stretch of undefined sites, $I^+_w[i, j](S_1)$ is now a gap, whereas $I^+_w[i, j](S_2) = I^+_p[i, j](S_0)$. Now suppose S_0 is extendable to an optimal solution A^+, which implies that $I^+_p[i, j](S_0) = A^+_p[i, j]$. Proposition 4 implies that either $A^+_w[i, j]$ is a gap, or $A^+_w[i, j] = A^+_p[i, j]$. If $A^+_w[i, j]$ is a gap, then $I^+_w[i, j](S_1) = A^+_w[i, j]$, meaning that S_1 is extendable to A^+. If $A^+_w[i, j] = A^+_p[i, j]$, then $I^+_w[i, j](S_2) = I^+_p[i, j](S_0) = A^+_p[i, j] = A^+_w[i, j]$, meaning that S_2 is extendable to A^+. Thus one of S_1 or S_2 remains extendable to A^+.

Now, without loss of generality we suppose S_1 is extendable to A^+ and prove that S_2 must be extendable to another optimal solution B^+. The proof that assuming S_2 is extendable to A^+ implies that S_1 is extendable to B^+ is symmetric.

Because $I^+_w[i, j](S_1)$ is a gap, then $A^+_w[i, j]$ must also be a gap. But then, by Lemma 6, there exists another optimal solution B^+ such that $B^+_w[i, j] = B^+_p[i, j]$ and such that $A^+_x[k] = B^+_x[k]$, whenever node $x \notin V(T_w)$ or $k \notin [i, j]$. Because $I^+(S_2)$ is such that $I^+_w[i, j](S_2) = I^+_p[i, j](S_0)$, and $I^+_x[k](S_2) = I^+_x[k](S_1)$ whenever $x \neq w$ or $k \notin [i, j]$, it is easy to see that S_2 is extendable to B^+. In detail,

- **for any node** $x \notin V(T_w)$, $I^+_x(S_2) = I^+_x(S_1)$ and $B^+_x = A^+_x$, so since here S_1 is extendable to A^+, then S_2 is extendable to B^+ for these nodes.

- **for node** w **and sites** $k \notin [i, j]$, $I^+_w[k](S_2) = I^+_w[k](S_1)$ and $B^+_w[k] = A^+_w[k]$, so since here S_1 is extendable to A^+, then S_2 is extendable to B^+ for these sites.

- **for node** w **and sites in** $[i, j]$, $I^+_w[i, j](S_2) = I^+_p[i, j](S_0) = A^+_p[i, j] = B^+_p[i, j] = B^+_w[i, j]$, so S_2 is identical (and thus extendable) to B^+ at these entries.

- **for** $x \in V(T_w) \setminus \{w\}$, because of the order with which the top-down phase (Algorithm 3) visits nodes, all the labeled gaps at x in S_2 are the same that have been set by the bottom-up phase (Algorithm 2). We can thus apply Proposition 1, which implies that the sites that are set to 1 in $I^+_x(S_2)$ are precisely those that equal 1 in all candidate solutions. Moreover by

Proposition 2, all sites that are set to 0 in $I_x^+(S_2)$ are also equal to 0 in any optimal solution. These two observations imply that in the descendants of w, S_2 is extendable to any optimal solution, and in particular to B^+.

This concludes the proof of point (b) above, and completes the entire proof. □

2 Correctness of the graph construction phase

Lemma 8. Suppose that $k \in [0, m+1]$ is not in a labeled gap at w at the end of the bottom-up phase. Then every path from 0 to $m+1$ in G_w must traverse k.

Proof. If there existed a path from 0 to $m+1$ in G_w not traversing k, then this path would have to traverse an arc (i, j) with $i < k < j$. But the only way an arc (i, j) between non-consecutive sites can be in G_w is because of the application of one of rules R3.1 – R3.3 to a labeled gap containing k, which leads to a contradiction. □

Theorem 2. Let $V \subseteq [0, m+1]$ and $w \in V(T) \setminus L(T)$.
V is the vertex set of a directed path from 0 to $m+1$ in G_w if and only if $\exists A^+$ optimal solution of the DPP such that $V = \mathbb{1}(A_w^+)$.

Proof. For the sake of conciseness, in this proof we use the following notation:

$$\mathcal{P}_w(i,j) = \{V \subseteq [i,j] : V \text{ is the vertex set of a path from } i \text{ to } j \text{ in } G_w\}.$$

Using the notation above, we prove a more general statement, which reduces to the Theorem's statement for $k = 0$, $k' = m+1$:

Claim. Suppose $k, k' \in [0, m+1]$ $(k \leq k')$ are not in any labeled gap at w at the end of the bottom-up phase. Then, $\forall V \subseteq [k, k']$:

$$V \in \mathcal{P}_w(k,k') \Leftrightarrow \exists A^+ \text{ optimal } : V = \mathbb{1}(A_w^+) \cap [k,k'].$$

We prove this Claim by double induction, over the depth of w and over the number of labeled gaps at w between k and k'. More precisely we prove the following statements which imply the Claim: (*base case 1*) the claim holds for $w = r(T)$; (*base case 2*) the claim holds for any node w when there are no labeled gaps between k and k'; finally, assuming that (*induction hypothesis 1*) the statement holds for p, the parent of w, and that (*induction hypothesis 2*) it holds for w if there are up to N labeled gaps between k and k', then (*induction step*) the statement holds for w if there are $N+1$ labeled gaps at w between k and k'.

(*Base case 2*): If there are no labeled gaps between k and k', then by construction G_w only has one path from k to k', the path that traverses every vertex in $[k,k']$. That is, $\mathcal{P}_w(k,k') = \{[k,k']\}$. Moreover, every solution is such that A_w^+ is only composed of 1s between k and k', by Proposition 1. That is, $\mathbb{1}(A_w^+) \cap [k,k'] = [k,k']$. Thus, $\forall V \subseteq [k,k']$, the following equivalences hold: $V \in \mathcal{P}_w(k,k') \Leftrightarrow V = [k,k'] \Leftrightarrow \exists A^+ \text{ optimal } : V = \mathbb{1}(A_w^+) \cap [k,k']$.

(*Base case 1*): If $w = r(T)$, then by Corollary 2 there can be no labeled gaps between k and k' and the proof of base case 1 applies.

Before proving the induction step, we need to prove 3 special cases, corresponding to the scenario where k and k' precisely delimit a labeled gap $[k+1, k'-1]$.

($[k+1, k'-1]$ *0-gap*):
If $[k+1, k'-1]$ is a 0-gap at w, we can prove the Claim as follows:
$V \in \mathcal{P}_w(k, k') \Leftrightarrow^{(1)} V = \{k, k'\} \Leftrightarrow^{(2)} \exists A^+ \text{ optimal } : V = \mathbb{1}(A^+_w) \cap [k, k']$.
Step (1) holds because by construction of G_w, it only has one path from k to k' and $\mathcal{P}_w(k, k') = \{\{k, k'\}\}$. Step (2) uses the fact that, by Proposition 2, every optimal A^+ must be such that $A^+_w[k+1, k'-1]$ is a gap, meaning that $\mathbb{1}(A^+_w) \cap [k, k'] = \{k, k'\}$.

($[k+1, k'-1]$ *P-gap*):
If $[k+1, k'-1]$ is a P-gap at w, then by construction, the subgraphs of G_w and G_p induced by $[k, k']$ are identical. We can prove the Claim as follows:
$V \in \mathcal{P}_w(k, k') \Leftrightarrow^{(1)} V \in \mathcal{P}_p(k, k') \Leftrightarrow^{(2)} \exists A^+ \text{ optimal} : V = \mathbb{1}(A^+_p) \cap [k, k'] \Leftrightarrow^{(3)}$
$\exists A^+ \text{ optimal} : V = \mathbb{1}(A^+_w) \cap [k, k']$.
Step (1) holds because $\mathcal{P}_w(k, k') = \mathcal{P}_p(k, k')$ by construction of G_w. Step (2) holds because by Corollary 3, k and k' are not in any labeled gap at p, meaning that we can apply induction hypothesis 1. Step (3) holds because $A^+_w[k, k'] = A^+_p[k, k']$ by Proposition 3.

($[k+1, k'-1]$ *C-gap*):
If $[k+1, k'-1]$ is a C-gap at w, then by construction the subgraphs of G_w and G_p induced by $[k, k']$ are identical, except for the addition of arc (k, k') to G_w. We can prove the Claim as follows:
$V \in \mathcal{P}_w(k, k') \Leftrightarrow^{(1)} V = \{k, k'\} \text{ or } V \in \mathcal{P}_p(k, k') \Leftrightarrow^{(2)}$
$V = \{k, k'\} \text{ or } \exists A^+ \text{ optimal} : V = \mathbb{1}(A^+_p) \cap [k, k'] \Leftrightarrow^{(3)}$
$\exists A^+ \text{ optimal} : V = \mathbb{1}(A^+_w) \cap [k, k']$.
Step (1) holds because $\mathcal{P}_w(k, k') = \mathcal{P}_p(k, k') \cup \{\{k, k'\}\}$ by construction of G_w. Step (2) holds again by Corollary 3 and the induction hypothesis 1. In step (3) the backward direction ($\Leftarrow^{(3)}$) holds because $\mathbb{1}(A^+_w) \cap [k, k']$ must be equal to one of $\{k, k'\}$ or $\mathbb{1}(A^+_p) \cap [k, k']$, by Proposition 4.

The forward direction ($\Rightarrow^{(3)}$) is slightly more complex. First suppose $V = \{k, k'\}$. In this case, since $\exists A^+$ optimal s.t. $A^+_w[k+1, k'-1]$ is a gap by Lemma 6, it means that $V = \{k, k'\} = \mathbb{1}(A^+_w) \cap [k, k']$. Now suppose $\exists A^+$ optimal s.t. $V = \mathbb{1}(A^+_p) \cap [k, k']$. In this case, since $\exists B^+$ optimal s.t. $B^+_w[k, k'] = B^+_p[k, k'] = A^+_p[k, k']$ by Lemma 6, it means that $V = \mathbb{1}(A^+_p) \cap [k, k'] = \mathbb{1}(B^+_w) \cap [k, k']$.

(*Induction step*):
Suppose there are $N+1$ labeled gaps at w between k and k', with $N \geq 0$. Let $[i, j]$ be the rightmost labeled gap among these $N+1$ labeled gaps, meaning that we can decompose $[k, k']$ as: $[k, i-1] \cup [i, j] \cup [j+1, k']$ where $[k, i-1]$ contains N labeled gaps, with both k and $i-1$ outside of these labeled gaps, and $[j+1, k']$ does not contain, nor intersect, with any labeled gap. We can prove the induction step as follows:
$V \in \mathcal{P}_w(k, k') \Leftrightarrow^{(1)}$
$V \cap [k, i-1] \in \mathcal{P}_w(k, i-1)$ and
$V \cap [i-1, j+1] \in \mathcal{P}_w(i-1, j+1)$ and
$V \cap [j+1, k'] \in \mathcal{P}_w(j+1, k') \Leftrightarrow^{(2)}$
$\exists B^+ \text{ optimal} : V \cap [k, i-1] = \mathbb{1}(B^+_w) \cap [k, i-1]$ and
$\exists C^+ \text{ optimal} : V \cap [i-1, j+1] = \mathbb{1}(C^+_w) \cap [i-1, j+1]$ and
$\exists D^+ \text{ optimal} : V \cap [j+1, k'] = \mathbb{1}(D^+_w) \cap [j+1, k'] \Leftrightarrow^{(3)}$

$\exists A^+$ optimal : $V \cap [k, k'] = \mathbb{1}(A_w^+) \cap [k, k']$.
Step (1) holds because every path from k to k' must traverse $i-1$ and $j+1$ by Lemma 8. Step (2) holds because:
$V \cap [k, i-1] \in \mathcal{P}_w(k, i-1) \Leftrightarrow \exists B^+$ optimal : $V \cap [k, i-1] = \mathbb{1}(B_w^+) \cap [k, i-1]$, by the induction hypothesis 2 (as there are N labeled gaps between k and $i-1$);
$V \cap [i-1, j+1] \in \mathcal{P}_w(i-1, j+1) \Leftrightarrow \exists C^+$ optimal : $V \cap [i-1, j+1] = \mathbb{1}(C_w^+) \cap [i-1, j+1]$, because as we have proven above the Claim is true when $k = i-1$ and $k' = j+1$ delimit a labeled gap;
$V \cap [j+1, k'] \in \mathcal{P}_w(j+1, k') \Leftrightarrow \exists D^+$ optimal : $V \cap [j+1, k'] = \mathbb{1}(D_w^+) \cap [j+1, k']$, by the base case 2 (as there are no labeled gaps between $j+1$ and k').
Finally, while step ($\Leftarrow^{(3)}$) is trivially true, step ($\Rightarrow^{(3)}$) can be proven by applying Lemma 7 twice: from B^+ and C^+ we can prove the existence of an optimal E^+ s.t. $E_w^+ = B_w^+[0, i-1]\, C_w^+[i, m+1]$, and from E^+ and D^+ we can prove the existence of an optimal F^+ s.t. $F_w^+ = E_w^+[0, j+1]\, D_w^+[j+2, m+1]$. (Recall Def. 7 for the concatenation of arrays of characters.) Noting that all solutions have 1s at sites $i-1$ and $j+1$ in w, all of the above implies

$$F_w^+[k, i-1] = B_w^+[k, i-1] \quad F_w^+[i-1, j+1] = C_w^+[i-1, j+1] \quad F_w^+[j+1, k'] = D_w^+[j+1, k'].$$

Thus F^+ is such that
$V \cap [k, i-1] = \mathbb{1}(B_w^+) \cap [k, i-1] = \mathbb{1}(F_w^+) \cap [k, i-1]$ and
$V \cap [i-1, j+1] = \mathbb{1}(C_w^+) \cap [i-1, j+1] = \mathbb{1}(F_w^+) \cap [i-1, j+1]$ and
$V \cap [j+1, k'] = \mathbb{1}(D_w^+) \cap [j+1, k'] = \mathbb{1}(F_w^+) \cap [j+1, k']$,
meaning that $V \cap [k, k'] = \mathbb{1}(F_w^+) \cap [k, k']$. Thus the Claim is verified with $A^+ = F^+$. This concludes the proof of step ($\Rightarrow^{(3)}$) and of the induction step. □

3 Complexity analysis

3.1 Algorithm 1

Remark 1. In all the proofs that follow, we assume the following data structure for each stored S: S is a map whose keys are the vertices in $V(T)$ and whose values S_u are lists of labeled gaps. Moreover, for each $u \in V(T)$, the list S_u is sorted by the indices of the labeled gaps, so that the labeled gaps with the smallest indices for their endpoints appear first.

Recall that b denotes the number of boundaries in the input alignment A:

$$b = |\{k \in [0, m] : A[k] \neq A[k+1]\}|.$$

It is easy to see that b is a very natural parameter to describe an alignment A. As we show in Lemma 9, the number b also arises when counting the number of blocks, and of breakpoints in A, defined as follows.

Given an alignment A for X, a *block* of A is a maximal interval $[i, j]$ such that $A[k]$ is the same $\forall k \in [i, j]$. Given a gap $A_x[i, j]$, we say that its *breakpoints* are real numbers i and $j+1$. The rationale behind the definition of breakpoint is that if we represent each site as a segments of length 1 over the real line (i.e., site 0 is the segment from 0.0 to 1.0, site 1 is the segment from 1.0 to 2.0, etc.) then a gap $[i, j]$ corresponds to the segment from i to $j+1$. Similarly, if $[i, j]$ is a labeled gap at node w, its breakpoints are real numbers i and $j+1$.

Lemma 9 (Characterization of b)**.** Let A be an alignment. The following propositions are equivalent:

1. A has b boundaries.
2. A has $b+1$ blocks.
3. $|\{x \in \mathbb{R} :$ there exists a gap in A which has x as a breakpoint$\}| = b$

Proof. The equivalence between points 1 and 2 is trivial.

As for the equivalence between points 1 and 3, this can be shown by noting that $(k, k+1)$ is a boundary if and only if $k+1$ is a breakpoint for some gap in A. The forward direction (boundary $\Rightarrow$ breakpoint) can be proven as follows: if k is such that $A[k] \neq A[k+1]$, then $\exists w \in L(T)$ such that $A_w[k] \neq A_w[k+1]$. But then two cases are possible: $A_w[k] = 1, A_w[k+1] = 0$, in which case $k+1$ is a breakpoint of a gap $[k+1, j]$ for some $j \geq k+1$; alternatively, $A_w[k] = 0, A_w[k+1] = 1$, in which case $k+1$ is a breakpoint of a gap $[i, k]$ for some $i \leq k$. The converse implication (breakpoint $\Rightarrow$ boundary) can be shown analogously. $\square$

Lemma 10. Let $w \in V(T)$. At the end of the bottom-up phase, each breakpoint of a labeled gap at w must be a breakpoint of some gap $A_x[i, j]$ for $x \in L(T_w)$.

Proof. By induction on the height of w. If w is a leaf, then any labeled gap $[i, j]$ is a 0-gap and thus corresponds to a gap $A_w[i, j]$ with the same breakpoints as $[i, j]$.

If w is an internal node, then the labeled gap $[i, j]$ must be obtained by the bottom-up phase as the intersection of labeled gaps $[i_u, j_u]$ at u and $[i_v, j_v]$ at v, where u and v are the children of w. It is easy to see that a consequence of intersecting two intervals is that each breakpoint of $[i, j]$ is either a breakpoint of $[i_u, j_u]$ or of $[i_v, j_v]$. But by the inductive hypothesis the breakpoints of $[i_u, j_u]$ and of $[i_v, j_v]$ are breakpoints of some gap in $L(T_u) \cup L(T_v) = L(T_w)$. $\square$

Lemma 11. Let $w \in V(T)$. The number of labeled gaps at w is at most $b/2$.

Proof. By Lemmas 9 and 10, any breakpoint of a given labeled gap must be one of the b breakpoints of some gap in A. Moreover, considering that the labeled gaps at w cannot overlap and cannot share a breakpoint (by Lemma 4), each breakpoint can be used at most once to create a labeled gap, and two breakpoints can create a labeled gap only if there is no other labeled gap between them. Thus, there are at most $\frac{b}{2}$ labeled gaps in w.

While in the argument above the number of labeled gaps is the one at the end of the bottom-up phase, the statement remains true for the number of 0-gaps at w during the top-down phase. This is because 0-gaps inherit their breakpoints from labeled gaps at their ancestors, meaning that any breakpoint of a 0-gap must be one of the b breakpoints of A. $\square$

Lemma 12. Assume that the input alignment is represented in gap form. Then the bottom-up phase runs in $O(nb)$ time.

Proof. We are going to show here that the bottom-up phase processes a single node $w \in V(T)$ in $O(b)$ time, which yields the statement.

First, if $w \in L(T)$, each gap in A_w is read and stored in S_w as a 0-gap in constant time (lines 2 to 4 in Algorithm 2). Since there at most $O(b)$ such gaps (by Lemma 11), any leaf is processed in $O(b)$ time.

If w is an internal node with children u and v, S_w can be efficiently computed from S_u and S_v in $O(b)$ time as follows (corresponding to line 9 in Algorithm 2). Initially $[i_u, j_u]$ is set to the first labeled gap in S_u and $[i_v, j_v]$ to the first labeled gap in S_v. If $[i_u, j_u] \cap [i_v, j_v] \neq \emptyset$, then we can add this intersection to S_w. Next, if $j_u < j_v$ then $[i_u, j_u]$ is reset so that it represents the next labeled gap in S_u, if $j_v < j_u$ then $[i_v, j_v]$ is reset so that it represents the next labeled gap in S_v, and if $j_u = j_v$ then both $[i_u, j_u]$ and $[i_v, j_v]$ are reset to next labeled gaps in their respective lists. We iterate this procedure until the end of one of the two lists is reached. Clearly, this procedure requires $O(|S_u| + |S_v|) = O(b)$ time. Note that $|S_w| = O(b)$ for any node w, because of Lemma 11. Moreover, the label for each labeled gap (line 11) is computable in constant time, giving a total of $O(b)$ time to process node w. □

Theorem 3. Assume that the input alignment A and every solution A^+ in output is represented in gap form. Then Algorithm 1 runs in $O(nbs)$ time.

Proof. We start by analysing the running time required to duplicate solutions throughout the top-down phase (as specified by rule R2.2). Note that although $|Sol|$ can grow during execution, it is bounded by s. To reach the final number of solutions s, there must be exactly $s-1$ duplications throughout the top-down phase. Creating a new copy of S can be done in $O(nb)$ time, since $O(nb)$ is also the space required to store a solution S (by Remark 1 and Lemma 11). Thus the total running time employed by duplications is $O(nbs)$.

We now consider the running time of the top-down phase (Algorithm 3 launched as TopDown$(r(T), Sol)$) *without* the duplication of solutions. We show that lines 3 – 9 inside the **for** $S \in Sol$ loop in Algorithm 1 can be executed in $O(b)$ time. For any fixed S, recall that by R2.2 some of its C-gaps at w are reset as 0-gaps, while some other C-gaps are replaced by 0-gaps at w's parent p. Resetting some of the C-gaps in S_w^C as 0-gaps requires $O(|S_w^C|) = O(b)$ time. Moreover, it is easy to see that copying the 0-gaps at p that fall within any P-gap at w, or within one of the remaining C-gaps in S_w^C, also requires $O(b)$ time in aggregate (because it can be done by scanning S_p once, with $|S_p| = O(b)$ by Lemma 11).

Repeating the operations above once for each $S \in Sol$ and once for each internal node gives a total running time for the top-down phase of $O(nbs)$, excluding solution duplications.

Now recall that because the input alignment is in gap form, the bottom-up phase runs in $O(nb)$ time by Lemma 12. Finally note that returning Sol, whose elements are also in gap form, can also be done in $O(nbs)$ time. □

3.2 Algorithm including the graph construction phase

Proposition 5. For any internal node w, G_w is a planar graph.

Proof. Let $G_w = ([0, m+1], E_w)$. Below, we prove that for any pair of arcs $(h_1, k_1), (h_2, k_2) \in E_w$ one of the following two cases must be verified:

1. One of the two arcs occurs "to the left" of the other, meaning that we have either $h_1 < k_1 \leq h_2 < k_2$ or $h_2 < k_2 \leq h_1 < k_1$.
2. The two arcs are "nested", meaning that either $[h_1, k_1] \subseteq [h_2, k_2]$ or $[h_1, k_1] \supseteq [h_2, k_2]$.

Clearly this implies the statement, as it means that drawing the graph with vertices $[0, m+1]$ along a line in their natural ordering involves no crossing arcs.

We prove the necessity of points 1 or 2 by induction on the depth of w. If $w = r(T)$ then $E_w = \{(k, k+1) : k \in [0, m]\}$, which implies that case 1 is necessarily verified. Now suppose that points 1 or 2 are verified for node p, where p is the parent of w. We show that one of them must also be true for w.

First consider the case where one of the two arcs connects consecutive sites, i.e. we can write $(h_1, k_1) = (i, i+1)$ without loss of generality. Let us consider the other arc. If $h_2 \geq i+1$ or $k_2 \leq i$ then clearly point 1 is verified. Otherwise we must have $h_2 < i+1$ and $k_2 > i$, which implies point 2.

Now suppose that neither of $(h_1, k_1), (h_2, k_2)$ connects consecutive sites. This implies that they both have been introduced by application of rules R3.1 – R3.3. Suppose that (h_1, k_1) was introduced when processing labeled gap $[i_1, j_1]$ and that (h_2, k_2) was introduced when processing labeled gap $[i_2, j_2]$. We now consider two cases: $[i_1, j_1] \neq [i_2, j_2]$ and $[i_1, j_1] = [i_2, j_2]$.

If $[i_1, j_1] \neq [i_2, j_2]$ then, by Lemma 4, these two labeled gaps cannot overlap, and they cannot cover consecutive sites (i.e. we cannot have $j_1 = i_2 - 1$ or $j_2 = i_1 - 1$). Without loss of generality let $[i_1, j_1]$ be to the left of $[i_2, j_2]$, i.e. $j_1 + 1 \leq i_2 - 1$. But then, since the rules require $h_1, k_1 \in [i_1 - 1, j_1 + 1]$ and $h_2, k_2 \in [i_2 - 1, j_2 + 1]$, we must have $h_1, k_1 \leq j_1 + 1 \leq i_2 - 1 \leq h_2, k_2$, which proves point 1 above.

Now suppose that both (h_1, k_1) and (h_2, k_2) were introduced when processing the same labeled gap $[i, j]$. If this is a 0-gap then by rule R3.1 we must have $(h_1, k_1) = (h_2, k_2) = (i-1, j+1)$ meaning that point 2 is verified. If $[i, j]$ is a P-gap, then $(h_1, k_1), (h_2, k_2) \in E_p$ where E_p is the arc set of G_p; but then either of points 1 or 2 must be verified, by the inductive hypothesis. Finally, if $[i, j]$ is a C-gap, then we can either have $(h_1, k_1), (h_2, k_2) \in E_p$, a case that we have just treated, or at least one of the two arcs must be equal to $(i-1, j+1)$, which implies that the two arcs are nested, i.e. point 2.

This concludes the proof by induction that for every pair of arcs in G_w either of points 1 or 2 holds, which implies that G_w is planar. □

Corollary 5. For any internal node w, the constructed graph $G_w = (V_w, E_w)$ is such that $|E_w| = O(m)$.

Proof. Because G_w is planar (Proposition 5), we can deduce with Euler's formula for graphs that $|E_w| \leq 3|V_w| - 6$. Thus, G_w cannot have more than $3(m+2) - 6 = 3m$ arcs. □

Theorem 4. The algorithm that first runs the bottom-up phase and then the graph construction phase runs in $O(nm)$ time.

Proof. By Lemma 12 the bottom-up phase runs in $O(nb)$ time.

As for the graph construction phase, we now show that it takes $O(m)$ time to construct a single graph $G_w = (V_w, E_w)$. Clearly V_w can be constructed in $O(m)$ time. Now consider the initialization of E_w via rule R3.0, which depends on finding all sites that are not contained in any labeled gap of S_w. This can also be done in $O(m)$ time by considering each site in turn; for each site k we only need to check whether k belongs to one of two labeled gaps: either the labeled

gap $[i,j]$ where we last found a previous site, or the labeled gap following $[i,j]$ in S_w.

Next, we need to add to E_w the arcs of rules R3.1, R3.2 and R3.3. It is easy to see that this can also be done in $O(m)$ time. One way to do this is to first consider all 0-gaps in S_w and apply rule R3.1 to each. Then we consider all P-gaps in S_w. Here by rule R3.2, we need to find all arcs $(h,k) \in E_p$ such that $h,k \in [i-1, j+1]$ for some P-gap $[i,j] \in S_w$. To do this, for each $h \in [0,m]$ in ascending order, we consider all outgoing arcs $(h,k) \in E_p$ and for each arc we only need to check whether h,k both belong to one of two P-gaps in S_w, similarly to what described in the last paragraph. Thus applying rule R3.2 to all P-gaps at once takes $O(|E_p|)$ time, which is $O(m)$ by Corollary 5. For the same arguments, applying rule R3.3 to all C-gaps takes $O(|E_p|) = O(m)$ time.

Thus, each graph is computed in $O(m)$ time. Since this must be repeated for all internal nodes, the graph construction phase requires $O(nm)$ time. Adding to this the bottom-up phase, the total complexity is $O(nb)+O(mn) = O(mn)$. □

4 Chindelevitch et al.'s algorithm for DPP

Here, we show that the algorithm for DPP shown in Chindelevitch et al. [1], transcribed in Algorithm 4, may fail to return an optimal solution. Note that Chindelevitch et al. denote a deletion from p to u at interval $[i,j]$ as the pair $(u, [i,j])$. Moreover the edge set and vertex set of tree T are denoted by E_T and V_T, respectively.

Algorithm 4: DeletionParsimony(Alignment A, tree T)

`// Transcribed from Chindelevitch et al. [1]`

Initialization:
for *every leaf* u **do**
  set $S_u = \{(i,j) | A_u(k) = 0 \ \forall i \leq k \leq j, A_u(i-1) = 1 = A_u(j+1)\}$
Recursion:
for *each internal node* $u \in V_T$*, in a post-order traversal* **do**
  Let v and w be the two children of u
  Set $R_v = \{(i,j) \in S_v | \exists (k,l) \in S_w \text{ with } (i,j) \subseteq (k,l)\}$
  Set $R_w = \{(i,j) \in S_w | \exists (k,l) \in S_v \text{ with } (i,j) \subseteq (k,l)\}$
  Set $S_u = (S_v - R_v) \cup (S_w - R_w)$
Termination:
Return $D = \bigcup_{u \in E_T} \bigcup_{(i,j) \in S_u - R_u} \{(u, [i,j])\}$.

Now consider the input depicted in Fig. S1, of a tree with 3 taxa, and an alignment of length 3 (+2 additional columns) of these taxa.

In the initialization, the algorithm computes a set S_u for each leaf:

$$S_a = \{(1,2)\}$$
$$S_b = \{(2,3)\}$$
$$S_c = \emptyset$$

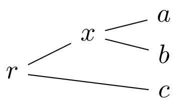

	**0**	**1**	**2**	**3**	**4**
a	1	0	0	1	1
b	1	1	0	0	1
c	1	1	1	1	1

Figure S1: Input where the algorithm for DPP given by Chindelevitch et al. [1] fails to return an optimal solution. The unique optimal solution for this instance has two deletions: one from x to a at $[1, 2]$ and the other from x to b at $[2, 3]$.

corresponding to the gaps at the leaves. Then, the recursion considers each internal node in post-order. When traversing node x, it sets

$$R_a = \{(i, j) \in S_a | \exists (k, l) \in S_b \text{ with } (i, j) \subseteq (k, l)\} = \emptyset$$
$$R_b = \{(i, j) \in S_b | \exists (k, l) \in S_a \text{ with } (i, j) \subseteq (k, l)\} = \emptyset$$
$$S_x = (S_a - R_a) \cup (S_b - R_b) = \{(1, 2); (2, 3)\}$$

Then, it considers the root r and sets

$$R_x = \{(i, j) \in S_x | \exists (k, l) \in S_c \text{ with } (i, j) \subseteq (k, l)\} = \emptyset$$
$$R_c = \{(i, j) \in S_c | \exists (k, l) \in S_x \text{ with } (i, j) \subseteq (k, l)\} = \emptyset$$
$$S_r = (S_x - R_x) \cup (S_c - R_c) = \{(1, 2); (2, 3)\}$$

Finally, let us consider the termination part, with the computing of D, that is the set of deletions found by the algorithm. Here line 11, as written in the published paper, sets

$$D = \bigcup_{u \in E_T} \bigcup_{(i,j) \in S_u - R_u} \{(u, [i, j])\}.$$

Note that $u \in E_T$ cannot be syntactically correct here, as sets S_u, R_u and deletions $(u, [i, j])$ are defined for a node u, not an edge. We suppose that the authors mean that u should be an internal node of T. Under this assumption,

$$D = \{(a, [1, 2]); (b, [2, 3]); (x, [1, 2]); (x, [2, 3])\}.$$

Clearly D is suboptimal, as the (unique) optimal solution consist of deletions $\{(a, [1, 2]); (b, [2, 3])\}$.

We note that a way to fix Algorithm 4 for the particular input in Fig. S1 is to replace line 9 with

$$\text{Set } S_u = R_v \cup R_w.$$

However, it is easy to check that the resulting algorithm fails to return an optimal solution for the example in Fig. S2.

We also considered other ways to fix Algorithm 4. For example, replacing line 11 with

$$D = \bigcup_{(u,v) \in E_T} \bigcup_{(i,j) \in S_u - R_v} \{(u, [i, j])\}$$

also gives no consistent solution.

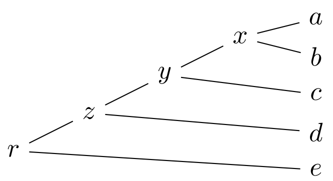

	0	1	2	3	4
a	1	0	0	1	1
b	1	1	0	0	1
c	1	1	0	1	1
d	1	1	0	1	1
e	1	1	1	1	1

Figure S2: Input where Algorithm 4 with line 9 replaced by "Set $S_u = R_v \cup R_w$" gives a suboptimal solution.

5 Solving the DPP with edge-specific costs

Consider the following problem.

Definition 8 (DPP WITH EDGE-SPECIFIC COSTS)**.**
Input: Tree T, alignment A for $L(T)$, and, for each edge $(u, v) \in E(T)$, cost $c_{uv} > 0$.
Output: A^+, an extension of A, minimizing the cost

$$c(A^+) = \sum_{(u,v) \in E(T)} c_{uv} \cdot d(A_u^+, A_v^+)$$

under the constraint that for all $(p, w) \in E(T)$ where p is the parent of w, all indels from p to w must be deletions.

In this problem each deletion has a cost that only depends on the edge where it occurs. This is useful to account for the fact that indel events are more likely to occur in some edges than others. Here we show how to modify the algorithm described in the main text, so as to solve this new problem.

The main idea is that the only thing that needs to be changed is the way labels 0, P and C are assigned to labeled gaps during the bottom-up phase. All other aspects of our work, including the graph-based representation of solutions, remain unchanged. In particular, if $[i, j]$ was a labeled gap at node w, $[i, j]$ remains a labeled gap in the new algorithm. Only its label may change. Later below we provide the pseudocode for the new bottom-up procedure (Algorithm 5) which replaces the old one (Algorithm 2). We do not provide a proof of correctness for this algorithm, but we describe the main ideas behind it and illustrate its functioning with an example.

5.1 Defining and computing $\text{cost}_w[i, j]()$

In order to assign the label of labeled gap $[i, j]$ at w, we need a few new definitions. Let $[i, j]$ be a labeled gap at node w. By Lemma 1, every leaf x descending from w must have a gap $A_x[i', j']$ such that $[i', j'] \supseteq [i, j]$. For brevity, we say that gap $A_x[i', j']$ *descends* from that labeled gap. Moreover, given a deletion from u to v at interval $[i, j]$, we say that it *covers* site $A_x[k]$ if $x \in L(T_v)$ and k is one of the sites where $A_u^+[k] = 1$ and $A_v^+[k] = 0$. Finally, given any set of deletions, its *total cost* is trivially defined as the sum of the costs of the deletions in it.

Definition 9. Let $[i, j]$ be a labeled gap at w. Assume $A_w^+[k] = 1$, $\forall k \notin [i, j]$.

- $\text{cost}_w[i,j](\neg\,\mathbb{0})$ is the minimum total cost of a set of deletions in T_w that cover all, and only, the sites in gaps descending from $[i,j]$.
- $\text{cost}_w[i,j](\mathbb{0})$ is the minimum total cost of a set of deletions in T_w that cover all, and only, the sites $A_x[k]$ in gaps descending from $[i,j]$, such that $k \notin [i,j]$.

The intuition behind Definition 9 is the following: $\text{cost}_w[i,j](\mathbb{0})$ is the minimum cost to pay to set to 0 all gaps descending from $[i,j]$, under the assumption that $A_w^+[i,j]$ is already filled with 0s. The $\mathbb{0}$ between the parentheses signifies that all sites in $[i,j]$ are assumed to be 0 at node w, and therefore also in any of its descendants $x \in V(T_w)$.

On the other hand, $\text{cost}_w[i,j](\neg\,\mathbb{0})$ is the minimum cost to pay to set to 0 all gaps descending from $[i,j]$, under the assumption that $A_w^+[i,j]$ is *not* filled with 0s. This explains the use of $\neg\,\mathbb{0}$ between the parentheses. Note that, by definition, $\text{cost}_w[i,j](\mathbb{0}) \le \text{cost}_w[i,j](\neg\,\mathbb{0})$.

Both quantities in Definition 9 can be computed recursively for all labeled gaps as follows. (We give a justification in the next subsection.) First, if w is a leaf, for every labeled gap $[i,j]$ at w:

$$\begin{aligned}\text{cost}_w[i,j](\mathbb{0}) &= 0\\ \text{cost}_w[i,j](\neg\,\mathbb{0}) &= +\infty\end{aligned} \tag{S3}$$

Now let w be an internal node with children u and v. Recall that any labeled gap $[i,j]$ at w is obtained as the intersection of labeled gaps at its children, *i.e.* there exist $[i_u,j_u]$ and $[i_v,j_v]$, labeled gaps at u and v respectively, such that $[i,j] = [i_u,j_u] \cap [i_v,j_v]$. Then,

$$\begin{aligned}\text{cost}_w[i,j](\neg\,\mathbb{0}) = \min&\begin{Bmatrix} c_{wu} + \text{cost}_u[i_u,j_u](\mathbb{0}),\\ \text{cost}_u[i_u,j_u](\neg\,\mathbb{0})\end{Bmatrix} +\\ \min&\begin{Bmatrix} c_{wv} + \text{cost}_v[i_v,j_v](\mathbb{0}),\\ \text{cost}_v[i_v,j_v](\neg\,\mathbb{0})\end{Bmatrix}\end{aligned} \tag{S4}$$

To compute $\text{cost}_w[i,j](\mathbb{0})$ we distinguish three cases. First, if $[i,j] = [i_u,j_u] = [i_v,j_v]$:

$$\text{cost}_w[i,j](\mathbb{0}) = \text{cost}_u[i,j](\mathbb{0}) + \text{cost}_v[i,j](\mathbb{0}). \tag{S5}$$

Second, if the two labeled gaps in w's children are strictly nested, that is, without loss of generality, if $[i,j] = [i_u,j_u] \subsetneq [i_v,j_v]$:

$$\text{cost}_w[i,j](\mathbb{0}) = \text{cost}_u[i,j](\mathbb{0}) + \min\begin{Bmatrix} c_{wv} + \text{cost}_v[i_v,j_v](\mathbb{0}),\\ \text{cost}_v[i_v,j_v](\neg\,\mathbb{0})\end{Bmatrix}. \tag{S6}$$

Third, if $[i_u,j_u]$ and $[i_v,j_v]$ partially overlap:

$$\text{cost}_w[i,j](\mathbb{0}) = \text{cost}_w[i,j](\neg\,\mathbb{0}). \tag{S7}$$

Remark that $\text{cost}_w[i,j](\neg\,\mathbb{0})$ has already been computed with Eqn. S4.

5.2 Justification for the recurrences.

We focus on Eqn. S6, as it illustrates well all aspects behind equations S3 – S7. Similar arguments to the one below can be made for each of those equations.

Recall that Eqn. S6 assumes that $[i, j]$ is a labeled gap at w and at its child u, and $[i_v, j_v] \supsetneq [i, j]$ is a labeled gap at the other child v. We want to calculate $\mathrm{cost}_w[i, j](\mathbb{0})$. Let D_w be an optimal set of deletions satisfying the requirements in Definition 9. That is, D_w is a minimum-cost set of deletions in T_w covering all and only the sites $A_x[k]$ in gaps descending from $[i, j]$, but not the sites with $k \in [i, j]$ because these are already filled with 0s.

Because $A_w^+[i, j]$ is filled with 0s, there cannot be any deletion in D_w from w to u at an interval inside $[i, j]$. Moreover, it is possible to show that any deletion from w to u at an interval *outside* $[i, j]$, i.e. not intersecting it or partially overlapping with it, would cover sites that are not in gaps that descend from the labeled gap $[i, j]$ at w. This is forbidden by Definition 9. So we can also exclude that D_w contains any deletion from w to u at an interval *outside* $[i, j]$.

Because no deletion in D_w can occur from w to u, we can partition D_w as

$$D_w = D_u \cup D_v \cup D_{wv}, \tag{S8}$$

where D_u contains the deletions of D_w occurring in T_u, D_v contains the deletions of D_w occurring in T_v, and D_{wv} contains the deletions occurring from w to v.

Let us now focus on D_{wv}. Recall that $[i_v, j_v]$ is a labeled gap at v. Any deletion from w to v at an interval outside $[i_v, j_v]$ would cover sites that are not in gaps that descend from labeled gap $[i, j]$, so by Definition 9, D_{wv} cannot contain any such deletion. Moreover there can be at most one deletion from w to v *inside* $[i_v, j_v]$, because two or more deletions at an interval within the same labeled gap would be suboptimal. All this means that $|D_{wv}| \leq 1$.

Going back to the partition in (S8), we can then write $\mathrm{cost}_w[i, j](\mathbb{0})$, which is the total cost of D_w, as the sum of the total costs of D_u, D_v and D_{wv}, where the total cost of D_{wv} is at most c_{wv}.

Recall that Eqn. S6 assumes that $A_w^+[i, j]$ is filled with 0s, which implies that the same holds for $A_u^+[i, j]$. Thus, D_u is a set of deletions that satisfies all the requirements in the definition of $\mathrm{cost}_u[i, j](\mathbb{0})$, and moreover it must have minimum total cost $\mathrm{cost}_u[i, j](\mathbb{0})$ (because otherwise D_w would not have minimum total cost itself). We have thus explained the first term in the right-hand side of Eqn. S6.

It remains to compute the total cost of $D_v \cup D_{wv}$. Here we must consider two cases: either D_{wv} contains a deletion from w to v that sets to 0 all sites in $[i_v, j_v]$, in which case the total cost of $D_v \cup D_{wv}$ is $c_{wv} + \mathrm{cost}_v[i_v, j_v](\mathbb{0})$, or $D_{wv} = \emptyset$, in which case we get $\mathrm{cost}_v[i_v, j_v](\neg\,\mathbb{0})$. Naturally, because $D_v \cup D_{wv}$ must be optimal, its cost must be the minimum between these two options, i.e. the 2nd term of Eqn. S6: $\min\{c_{wv} + \mathrm{cost}_v[i_v, j_v](\mathbb{0}), \mathrm{cost}_v[i_v, j_v](\neg\,\mathbb{0})\}$.

5.3 Assigning labels on the basis of $\mathrm{cost}_w[i, j](\,)$

For any labeled gap $[i, j]$ at w, once we have computed $\mathrm{cost}_w[i, j](\mathbb{0})$ and $\mathrm{cost}_w[i, j](\neg\,\mathbb{0})$, we can define its label as follows, where p is the parent of w:

$$\mathrm{label}_w[i, j] = \begin{cases} 0 & \text{if } c_{pw} + \mathrm{cost}_w[i, j](\mathbb{0}) < \mathrm{cost}_w[i, j](\neg\,\mathbb{0}), \\ P & \text{if } c_{pw} + \mathrm{cost}_w[i, j](\mathbb{0}) > \mathrm{cost}_w[i, j](\neg\,\mathbb{0}), \\ C & \text{if } c_{pw} + \mathrm{cost}_w[i, j](\mathbb{0}) = \mathrm{cost}_w[i, j](\neg\,\mathbb{0}). \end{cases} \tag{S9}$$

Algorithm 5 shows how all the ideas presented here can be integrated in a new bottom-up phase.

5.4 A small example

Consider the small example in Fig. S3. We use it to illustrate: (1) the definition of $\text{cost}_w[i,j](X)$, (2) the recurrences, (3) the label assignment.

To illustrate the definition of $\text{cost}_w[i,j](X)$, consider labeled gap $[3,3]$ at node x. Its descendant gaps are $A_b[3,3], A_c[3,3], A_d[1,3]$. Case $X = \mathbb{0}$: if we assume that $A_x^+[3,3] = 0$, then sites $A_b[3], A_c[3]$ and $A_d[3]$ do not need to be covered; $\text{cost}_x[3,3](\mathbb{0})$ is the minimum total cost of a set of deletions in T_x that cover the remainder of the sites in the descendant gaps, that is, $A_d[1,2]$. To cover them, we just need a single deletion from y to d, which gives $\text{cost}_x[3,3](\mathbb{0}) = c_{yd} = 1$. Case $X = \neg\,\mathbb{0}$: $\text{cost}_x[3,3](\neg\,\mathbb{0})$ is the minimum total cost of a set of deletions in T_x that cover exactly the sites in gaps $A_b[3,3], A_c[3,3], A_d[1,3]$. To do this, it is optimal to take deletion $[3,3]$ from x to b (cost 2), deletion $[3,3]$ from x to y (cost 1), and deletion $[1,2]$ from y to d (cost 1). Hence, $\text{cost}_x[3,3](\neg\,\mathbb{0}) = 4$.

We now consider each labeled gap and show how $\text{cost}_w[i,j](X)$ is actually computed for each of them. First, Eqn. S4 sets

$$\text{cost}_y[1,1](\neg\,\mathbb{0}) = \min\{2+0, \infty\} + \min\{1+0, \infty\} = 3,$$

and Eqn. S6 sets

$$\text{cost}_y[1,1](\mathbb{0}) = 0 + \min\{1+0, \infty\} = 1.$$

The calculation for $\text{cost}_y[3,3](X)$ is exactly the same as the one above for $\text{cost}_y[1,1](X)$. Finally, Eqn. S4 sets

$$\text{cost}_x[3,3](\neg\,\mathbb{0}) = \min\{2+0, \infty\} + \min\{1+1, 3\} = 4,$$

and Eqn. S5 sets

$$\text{cost}_x[3,3](\mathbb{0}) = \text{cost}_b[3,3](\mathbb{0}) + \text{cost}_y[3,3](\mathbb{0}) = 0 + 1 = 1.$$

Finally, the labels of both labeled gaps at y are set to 0, because $c_{xy} + 1 < 3$, and the label of $[3,3]$ at x is set to C, because $c_{rx} + 1 = 4$. Note that this is very different to what we would obtain for the standard (constant costs) DPP, where we would assign label C to the labeled gaps at y (by rule R1.4) and label 0 to $[3,3]$ at x (by rule R1.1).

In the end, it is easy to check that the computed labels result in the two solutions such that $A_x^+[3,3] \in \{0,1\}$ and $A_y^+[1,1] = A_y^+[3,3] = 0$.

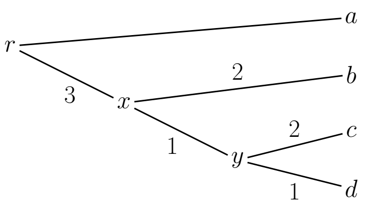

	**0**	**1**	**2**	**3**	**4**
a	1	1	1	1	1
x	1	1	1	$\mathbb{0}$: 1, $\neg\,\mathbb{0}$: 4 $\Rightarrow$ C	1
b	1	1	1	0	1
y	1	$\mathbb{0}$: 1, $\neg\,\mathbb{0}$: 3 $\Rightarrow$ 0	1	$\mathbb{0}$: 1, $\neg\,\mathbb{0}$: 3 $\Rightarrow$ 0	1
c	1	0	1	0	1
d	1	0	0	0	1

Figure S3: Resolution of a small instance of Problem 8. Left: input tree where cost c_{uv} labels edge (u,v). Right: table representation of A^+ showing labeled gaps as grey rectangles. Trivial labeled gaps (those at the leaves) are not shown for simplicity. For every other labeled gap, we report $\text{cost}_w[i,j](\mathbb{0})$ at the top of the rectangle, and $\text{cost}_w[i,j](\neg\,\mathbb{0})$ at the bottom. The assigned label appears to the right (after $\Rightarrow$).

Algorithm 5: NewBottomUp(w, A, S)

Data: w, a node of the binary tree T; A, an alignment for $L(T)$;
S, a data structure containing a set S_u of labeled gaps at u, $\forall u \in V(T)$;
cost, a data structure to retrieve the values of $\text{cost}_u[i,j](\,)$, $\forall [i,j] \in S_u$
Result: S_z and cost_z are filled $\forall z \in V(T_w)$
// recall that here $S_u = \emptyset, \forall u \in V(T_w)$
if *w is a leaf* **then**
  $S_w \leftarrow$ gaps in A_w
  for $[i,j] \in S_w$ **do**
    $\text{cost}_w[i,j](\mathbb{0}) = 0$
    $\text{cost}_w[i,j](\neg\,\mathbb{0}) = +\infty$
    assign label 0 to $[i,j]$
else
  Let u, v be the two children of w
  BottomUp(u, A, S)
  BottomUp(v, A, S)
  $S_w \leftarrow \{[i_u, j_u] \cap [i_v, j_v] : ([i_u, j_u], [i_v, j_v]) \in S_u \times S_v\} \setminus \{\emptyset\}$
  for $[i,j] \in S_w$ **do**
    Compute $\text{cost}_w[i,j](\neg\,\mathbb{0})$ using Eqn. S4
    Compute $\text{cost}_w[i,j](\mathbb{0})$ using one of Eqns. S5, S6, S7
    Let p be the parent of w
    $\Delta = c_{pw} + \text{cost}_w[i,j](\mathbb{0}) - \text{cost}_w[i,j](\neg\,\mathbb{0})$
    $L = \begin{cases} 0 & \text{if } \Delta < 0 \\ \text{P} & \text{if } \Delta > 0 \\ \text{C} & \text{if } \Delta = 0 \end{cases}$
    Assign label L to $[i,j]$

5.5 Justification for Eqn. S9.

Lemma 13. Let A^+ be an optimal solution for Problem 8. Let $[i, j]$ be a labeled gap at node w. Let $D_w[i, j]$ be the set of deletions in A^+ occurring in T_w and only covering sites in gaps descending from $[i, j]$.

$$\text{The total cost of } D_w[i,j] \; = \begin{cases} \text{cost}_w[i,j](\mathbb{0}) & \text{if } A^+_w[i,j] \text{ is a gap,} \\ \text{cost}_w[i,j](\neg\,\mathbb{0}) & \text{otherwise.} \end{cases}$$

We do not provide a proof for Lemma 13. The example in Figure S3 illustrates it well. Consider the optimal solution A^+ with $A^+_x[3,3] = 1$ and $A^+_y[1,1] = A^+_y[3,3] = 0$. For this solution, $D_x[3,3]$ contains three deletions: one from x to b at site 3, one from x to y at site 3, and one from y to d at site 2. Note that $D_x[3,3]$ *does not* contain any deletion covering site 1. The total cost of $D_x[3,3]$ equals 4, which is precisely $\text{cost}_x[3,3](\neg\,\mathbb{0})$, as predicted by Lemma 13.

Now let $[i, j]$ be a labeled gap at node w and let

- $D_w[i, j]$ be defined as in Lemma 13;
- $D_{pw}[i, j]$ be the set of deletions in A^+ from p to w, at an interval contained in $[i, j]$.

For the sake of argument let us momentarily assume that $A^+_p[i,j]$ is not a gap. In this case, by Lemma 13, the total cost of $D_w[i,j] \cup D_{pw}[i,j]$ is:

$$\begin{aligned} &= c_{pw} + \text{cost}_w[i,j](\mathbb{0}) && \text{if } A^+_w[i,j] \text{ is a gap,} \\ &= \text{cost}_w[i,j](\neg\,\mathbb{0}) && \text{if } A^+_w[i,j] = A^+_p[i,j], \\ &\geq c_{pw} + \text{cost}_w[i,j](\neg\,\mathbb{0}) && \text{otherwise.} \end{aligned} \tag{S10}$$

Because A^+ is optimal, the total cost of $D_w[i,j] \cup D_{pw}[i,j]$ must be equal to the minimum across the three choices in Eqn. S10. Now let us consider three cases on the basis of the possible (in)equalities between $c_{pw} + \text{cost}_w[i,j](\mathbb{0})$ and $\text{cost}_w[i,j](\neg\,\mathbb{0})$.

If $c_{pw} + \text{cost}_w[i,j](\mathbb{0}) < \text{cost}_w[i,j](\neg\,\mathbb{0})$, then the minimum of the three choices in Eqn. S10 is always the one in the first row. This means that, if $A^+_p[i,j]$ is *not* a gap, then $A^+_w[i,j]$ must be a gap. If, on the other hand, $A^+_p[i,j]$ is a gap, then by Observation 1, $A^+_w[i,j]$ is also a gap. Thus, if the $<$ relation holds in Eqn. S9, $A^+_w[i,j]$ must be a gap in any case, which justifies the fact that we assign the label 0 to it.

Similarly, if $c_{pw} + \text{cost}_w[i,j](\mathbb{0}) > \text{cost}_w[i,j](\neg\,\mathbb{0})$, then the minimum of the three choices in Eqn. S10 is always the one in the second row. This means that, if $A^+_p[i,j]$ is *not* a gap, then $A^+_w[i,j] = A^+_p[i,j]$. Clearly, this is also true if $A^+_p[i,j]$ is a gap. Since in any case $A^+_w[i,j] = A^+_p[i,j]$, this justifies the fact that if the $>$ relation holds in Eqn. S9, we assign label P.

Finally, it is intuitive that if $c_{pw} + \text{cost}_w[i,j](\mathbb{0}) = \text{cost}_w[i,j](\neg\,\mathbb{0})$, then both setting $A^+_w[i,j]$ as a gap or setting $A^+_w[i,j] = A^+_p[i,j]$ lead to an optimal solution, which justifies the assignment of label C in the third case of Eqn. S9. Note that the minimum of the three choices in Eqn. S10 can never be the one in the third row.

References

[1] Chindelevitch, L., Li, Z., Blais, E., and Blanchette, M. "On the inference of parsimonious indel evolutionary scenarios". In: *Journal of Bioinformatics and Computational Biology* 04.03 (2006), pp. 721–744.
